# Unveiling the link: Evaluating *MTHFR* gene polymorphisms and colorectal cancer risk through meta-analysis

**DOI:** 10.1371/journal.pone.0305517

**Published:** 2025-07-16

**Authors:** Yu-wei Wang, Ze-yi Huang, Chen-xue Jin, Xiao-hui Shen, Xiao-feng He, Chang-qing Yang

**Affiliations:** 1 Department of Digestive internal medicine, Heping Hospital Affiliated to Changzhi Medical College, Changzhi, Shanxi, China; 2 Changzhi Medical College, Changzhi, Shanxi Province, China; 3 Institute of Evidence-Based Medicine, Heping Hospital Affiliated to Changzhi Medical College, Changzhi, Shanxi Province, China; Noakhali Science and Technology University Faculty of Pharmacy, BANGLADESH

## Abstract

Colorectal cancer pathogenesis is a multifactorial process, with genetic factors playing a significant role in cancer development. A review of published meta-analyses on *MTHFR* gene polymorphisms and colorectal cancer susceptibility showed inconsistent findings and failed to assess the reliability of statistically significant results. Case-control studies were manually searched in databases to investigate the association between *MTHFR* gene polymorphisms and colorectal cancer. The study assessed the strength of association for the five gene models by calculating odds ratios (*ORs*) and 95% confidence intervals (*CIs*). The study results were also analyzed for the source of heterogeneity, sensitivity, publication bias, and false-positive report probability (FPRP) test. Additionally, extensive subgroup analyses were conducted to investigate the impact of confounding factors on the associations. The study suggests that *MTHFR C677T* gene polymorphism reduces the risk of colorectal cancer in Asian and mixed-race populations, while increasing the risk of Colorectal cancer in the Indian ethnic group. *MTHFR A1298C* may play a protective role in the development of colorectal cancer. These findings provide valuable insights for the early diagnosis and prevention of Colorectal cancer. However, further studies are required to confirm the association, which may offer additional information for the early diagnosis and prevention of Colorectal cancer.

## Introduction

Colorectal cancer (CRC) is a significant threat to human life and health, with the fourth-highest mortality rate and the third-highest incidence rate of all malignant tumors globally [1]. It poses a considerable burden to society [2]. The incidence of colorectal cancer (CRC) has been steadily increasing in recent years. According to the latest data, more than 1.9 million new cases of CRC were reported globally in 2020, with a mortality rate of 50%. This makes CRC the third most common malignant tumour among adults [3]. However, the pathogenesis of CRC remains poorly understood. The pathogenesis of CRC is a complex multifactorial process, as revealed by epidemiological studies. This process involves the interaction of genetic, epigenetic, and environmental factors. Genetic factors, such as single nucleotide polymorphisms (SNPs) in CRC-associated genes, as well as non-genetic factors, such as obesity, physical inactivity, smoking, and alcohol consumption, have been shown to play a significant role in the development of CRC [4,5]. In recent years, research has identified numerous genetic polymorphisms associated with CRC [6]. These SNPs can serve as markers for improving cancer diagnosis and determining therapeutic options [7]. Notably, the role of the folate metabolising enzyme gene - methylenetetrahydrofolate reductase (*MTHFR*) - in the pathogenesis of colorectal cancer has gained widespread attention among researchers[13].

*MTHFR* is a crucial enzyme in the folate metabolism pathway. It catalyses the irreversible conversion of 5,10-methylenetetrahydrofolate to 5-methyltetrahydrofolate, which regulates intracellular folate content and DNA methylation [8,9]. *MTHFR* has several SNPs loci in the clinic, of which C677T (RS1801133) and A1298C (RS1801131) are the two most significant. RS1801133 is located in exon 4 and converts cytosine (C) to thymine (T) at nucleotide 677. This prompts the conversion of alanine to valine at position 222, with three genotypes CC, CT and TT [10]. RS1801131, located in exon 7, drives the conversion of adenine (A) to cytosine (C) at nucleotide 1298, causing a glutamate to alanine mutation with genotypes AA, AC and CC [11]. Mutations in metabolic enzyme genes can interfere with the folate metabolic pathway, leading to folate deficiency. This is significant because folate plays a crucial role in DNA synthesis and biosynthesis of nucleotide precursors for DNA, RNA and protein methylation [12]. Mutations in the *MTHFR* gene can also cause DNA hypomethylation, resulting in aberrant expression of proto-oncogenes and alterations in gene transcription, which can promote CRC [13].

Numerous studies have demonstrated an association between *MTHFR* gene polymorphisms and various diseases, including psychiatric disorders, gastric cancer, hepatocellular carcinoma, lung cancer, cervical cancer, hypertension, cerebral infarction, and coronary heart disease [14]. However, the role of *MTHFR* gene polymorphisms in the pathogenesis of CRC remains inconclusive. This may be due to differences in study design, geography, ethnicity, and dietary habits. After reviewing previous meta-analyses [15–41] and basic studies [17,42–104] that explored the relationship between *MTHFR* gene polymorphisms and CRC susceptibility, it is evident that the results are still highly controversial. Issues such as delayed updating, inappropriate inclusion of literature, inconsistent quality of reporting, and omission of important literature may have contributed to this situation. Furthermore, prior meta-analyses have failed to evaluate the reliability of statistical associations, potentially resulting in erroneous conclusions. It is worth noting that a number of recent studies have been published [136–140]. Therefore, conducting more comprehensive and detailed meta-analyses based on existing case-control and cohort studies is necessary to clarify the correlation between the *MTHFR C677T* and *A1298C* polymorphisms and the risk of CRC. This will provide a population-based reference for CRC risk assessment, prevention, and control.

## Materials and methods

### Search strategy

The literature search was conducted in databases such as PubMed, Web of Knowledge, ISI, China Knowledge, and Wanfang databases in strict accordance with PRISMA criteria [141]. The search was confirmed by screening titles, abstracts, and reading the complete literature in detail. References to identified meta-analyses and reviews were also checked to ensure no relevant studies were missed. The deadline for the search is October 2023. The search strategy for the English database specifically includes the terms ‘polymorphism’, ‘variant’, ‘variation’, ‘mutation’, ‘SNP’, ‘genome-wide association study’, ‘genetic association study’, ‘genotype’, and ‘allele’, combined with the terms ‘colorectal’, ‘rectal’, ‘colon’, ‘intestine’, and ‘gut’, as well as ‘*MTHFR*’, ‘Methylenetetrahydrofolate reductase’, and ‘5, 10-Methylenetetrahydrofolate reductase’. The Chinese database was searched using the following subject terms: ‘methylenetetrahydrofolate reductase ‘, ‘gene polymorphism’, ‘colorectal cancer’, ‘rectal cancer’.

### Selection criteria

Inclusion criteria: (1) Case-control studies or cohort studies; (2) Correlation studies on the relationship between polymorphisms in the *MTHFR C677T* (*RS1801133*) and/or *A1298C* (*RS1801131*) genes and susceptibility to colon cancer, rectal cancer, and colorectal cancer; (3) The genotype frequencies corresponding to the genetic polymorphisms in the case and control groups can be obtained from the literature.

Exclusion criteria: (1) Studies with missing or duplicated data; (2) Reviews, letters, and case reports; (3) Studies that included colonic and rectal polyps in the case group; (4) Studies with poorly described data.

#### Data extraction.

To ensure the accuracy of the extracted information, two authors independently performed the inclusion and exclusion criteria for each study to extract useful information. In case of disagreement, the corresponding author would be responsible for re-extracting the data, which would then be confirmed and validated. Furthermore, when the data was insufficient or uncertain, the original authors were contacted to verify and supplement its accuracy. Studies with incomplete data were excluded, and only the highest quality studies were retained, while repetitive, duplicated, or similar studies were excluded. The provided information was used to complete a standardised table. This included the last name of the first author, year of publication, country, ethnicity (Asian, Caucasian, African, Indian, Mixed), sample size of cases and controls, source of controls (hospital-based or population-based), type of match, site of tumour, and frequency of genotypes in the cases and controls. The details can be found in Table 1.

**Table 1:** Characteristics of studies included in the meta-analysis.

First author/Year	Country	Geographic region	Ethnicity	Sample size	SC	Type of control	Matching	Tumour position	Genotypes distribution of *MTHFR C677T(rs1801133)*						HWE	Quality score	Genotypes distribution of *A1298C (rs1801131)*						HWE	Quality score
									Cases			Controls					Cases			Controls				
									CC	CT	TT	CC	CT	TT			AA	AC	CC	AA	AC	CC		
Chen et al. [42] 1996	USA	North American	Caucasian	144/627	PB	Non-gastric cancer	Age and sex	CR0043	67	64	13	280	263	84	0.0787	14	–	–	–	–	–	–	–	–
Ma J et al. [43] 1997	USA	North American	Caucasian	202/326	PB	Non-gastric cancer	Age and sex	CRC	92	92	18	145	132	49	0.0408	13	–	–	–	–	–	–	–	–
Park et al. [44] 1999	Korea	Asia	Asians	200/460	HB	Healthy controls	NR	CRC	65	107	28	140	246	74	0.0484	8	–	–	–	–	–	–	–	–
Slattery et al. [45] 1999	USA	North American	mixed	1467/1821	PB	Non-gastric cancer	Age and sex	colon	673	655	139	827	787	207	0.3412	14	–	–	–	–	–	–	–	–
Slattery et al. [46] 2000	USA	North American	mixed	232/164	PB	Non-gastric cancer	Age and sex	colon	106	107	19	73	71	20	0.6710	14	–	–	–	–	–	–	–	–
Ryan et al. [47] 2001	Ireland	European	Caucasian	136/848	HB	Healthy controls	NR	CRC	49	73	14	439	326	83	0.0523	10	–	–	–	–	–	–	–	–
Chen et al. [48] 2002	USA	North American	Caucasian	202/326, 210/344	PB	Non-gastric cancer	Age and sex	CRC	92	92	18	145	132	49	0.0408	12	95	98	17	153	159	32	0.2307	14
Sachse et al. [49] 2002	UK	European	Caucasian	490/592	HB	Non-gastric cancer	Age and sex	CRC	238	199	53	271	272	49	0.0918	12	–	–	–	–	–	–	–	–
Matsuo et al. [50] 2002	Japan	Asia	Asians	142/241, 141/241	HB	Non-gastric cancer	Age and sex	CRC	39	81	22	81	124	36	0.3039	13	94	44	3	157	75	9	0.9908	13
Shannon et al. [51] 2002	Australian	European	Caucasian	501/1207	HB	Healthy controls	Age	CRC	249	197	55	533	560	114	0.0557	10	–	–	–	–	–	–	–	–
Le Marchand et al. [52] 2002(a)	USA	North American	Asia	322/397, 315/395	PB	Non-gastric cancer	Age and sex	CRC	126	153	43	138	182	77	0.2246	15	205	100	10	244	136	15	0.4594	15
Le Marchand et al. [52] 2002 (b)	USA	North American	Caucasian	149/171, 148/171	PB	Non-gastric cancer	Age and sex	CRC	66	64	19	66	81	24	0.9147	15	78	56	14	86	65	20	0.1630	15
Keku et al. [53] 2002 (a)	USA	North American	African	244/329,243/329	PB	Non-gastric cancer	Age and sex	colon	198	43	3	264	59	6	0.2140	15	157	78	8	217	99	13	0.6864	15
Keku et al. [53] 2002 (b)	USA	North American	Caucasian	308/539,309/541	PB	Non-gastric cancer	Age and sex	colon	144	140	24	265	223	51	0.6812	15	156	132	21	237	236	68	0.4400	15
Huang et al. [54] 2003	Chongqing	Asia	Asians	82/82	HB	Non-gastric cancer	Age and sex	CRC	36	40	6	40	33	9	0.5813	7	–	–	–	–	–	–	–	–
Heijmans et al. [55] 2003	Netherlands	European	Caucasian	18/793	PB	NR	Age and sex	CRC	7	7	4	399	329	65	0.8064	13	–	–	–	–	–	–	–	–
Toffoli et al. [56] 2003	Italy	European	Caucasian	276/279	HB	Healthy controls	Age and sex	colon	93	145	38	83	140	56	0.8272	13	122	129	25	133	121	25	0.7350	13
Plaschke et al. [57] 2003	Germany	European	Caucasian	287/346	PB	Healthy controls	NR	CRC	133	120	34	149	159	38	0.6484	13	134	124	29	154	151	41	0.6694	13
Pufulete et al. [58] 2003	UK	European	Caucasian	28/76	HB	Non-gastric cancer	Age and sex	CRC	16	6	6	41	29	6	0.7841	13	16	10	2	47	26	3	0.7993	13
Curtin et al. [59] 2004	USA	North American	mixed	1608/1972	PB	Non-gastric cancer	Age and sex	colon	734	724	150	887	858	227	0.3733	14	757	698	153	929	827	216	0.1188	14
Yin et al. [60] 2004	Japan	Asia	Asians	685/778	PB	Non-gastric cancer	Age and sex	CRC	270	330	85	278	367	133	0.5284	15	438	220	27	515	244	19	0.1133	15
Ulvik et al. [61] 2004	Norway	European	Caucasian	2159/2190	PB	NR	Age and sex	CRC	1103	899	157	1092	886	212	0.1008	12	–	–	–	–	–	–	–	–
Kim et al. [62] 2004	Korea	Asia	Asians	243/225	HB	Non-gastric cancer	Age and sex	CRC	86	122	35	83	109	33	0.7731	14	–	–	–	–	–	–	–	–
Otani et al. [63] 2005	Japan	Asia	Asians	106/222, 106/224	HB	Healthy controls	Age and sex	CRC	32	49	25	51	114	57	0.6790	15	73	32	1	156	63	5	0.6425	15
Le Marchand et al. [64] 2005	USA	North American	mixed	817/2021	PB	Non-gastric cancer	Age and sex	CRC	394	336	87	987	779	255	0	13	–	–	–	–	–	–	–	–
Landi et al. [65] 2005	Spanish	European	Caucasian	350/309, 360/319	HB	Non-gastric cancer	Age and sex	CRC	128	158	64	109	139	61	0.1699	13	189	146	25	170	127	22	0.7937	13
Matsuo et al. [66] 2005	Japan	Asia	Asians	256/771, 257/767	HB	Non-gastric cancer	Age and sex	CRC	106	114	36	289	348	134	0.0999	14	163	85	9	479	257	31	0.6345	14
He MJ et al. [67] 2005	Guangdong	Asia	Asians	56/143	HB	Healthy controls	Age and sex	rectum	–	–	–	–	–	–	–	–	38	13	5	98	35	10	0.0112	9
Miao et al. [68] 2005	Beijing	Asia	Asians	198/420	HB	Healthy controls	Age and sex	CRC	53	87	58	133	201	86	0.5290	15	147	48	3	282	132	6	0.0293	13
Jiang Q et al. [69] 2005	Zhejiang	Asia	Asians	125/339, 124/335	PB	Non-gastric cancer	Age and sex	CRC	51	59	15	134	143	62	0.0320	13	93	30	1	226	103	6	0.1370	15
Wang et al. [70] 2006	India	Asia	Indian	302/291	HB	Non-gastric cancer	Age and sex	CRC	257	43	2	255	36	0	0.2607	13	141	130	31	105	135	51	0.5050	13
Van Guelpen et al. [71] 2006	Sweden	European	Caucasian	220/415, 220/412	PB	Non-gastric cancer	Age and sex	CRC	123	85	12	212	161	42	0.1682	14	85	103	32	189	173	50	0.2882	14
Battistelli et al. [72] 2006	Italy	European	Caucasian	93/100	HB	Healthy controls	Age and sex	CRC	32	40	21	30	51	19	0.7452	8	–	–	–	–	–	–	–	–
Koushik et al. [73] 2006	USA	North American	Caucasian	349/794	PB	Non-gastric cancer	Age and sex	CRC	166	145	38	355	327	112	0.0102	16	–	–	–	–	–	–	–	–
Webb et al. [74] 2006	UK	European	Caucasian	2556/2692	PB	Non-gastric cancer	Age and sex	CRC	1135	1161	260	1173	1193	326	0.3981	15	–	–	–	–	–	–	–	–
Liang et al. [75] 2006	Guangdong	Asia	Asians	56/143	HB	Healthy controls	Age and sex	rectum	33	15	8	90	50	3	0.1874	12	38	13	5	98	35	10	0.0112	10
Song et al. [76] 2006	Zhejiang	Asia	Asians	204/409,197/411	PB	Non-gastric cancer	Age and sex	CRC	75	102	27	174	174	61	0.1109	17	133	62	2	265	140	6	0.0084	15
Hubner et al. [77] 2007	UK	European	Caucasian	1685/2692	PB	Non-gastric cancer	NR	CRC	743	759	183	1173	1193	326	0.3981	12	–	–	–	–	–	–	–	–
Curtin et al. [78] 2007	USA	North American	mixed	916/1972	PB	Non-gastric cancer	Age and sex	CRC	432	402	82	887	858	227	0.3733	16	435	394	87	929	827	216	0.1188	16
Jin et al. [79] 2007	Zhejiang	Asia	Asians	449/672	HB	Healthy controls	Age and sex	CRC	182	211	56	211	325	136	0.5944	14	–	–	–	–	–	–	–	–
Murtaugh et al. [80] 2007	USA	North American	mixed	742/970	PB	Non-gastric cancer	Age and sex	rectum	357	301	84	466	392	112	0.0354	14	360	317	65	436	424	110	0.6526	16
Chang et al. [81] 2007	China	Asia	Asians	195/195	HB	Healthy controls	Age and sex	CRC	85	86	24	92	87	16	0.4667	14	120	65	10	127	55	13	0.0458	12
Lima et al. [82] 2007 (a)	Brazilian	South American	Caucasian	90/300	HB	Non-gastric cancer	Age and sex	CRC	36	40	14	143	127	30	0.8171	14	61	24	5	191	93	16	0.2970	14
Lima et al. [82] 2007 (b)	Brazilian	South American	African	10/300	HB	Non-gastric cancer	Age and sex	CRC	4	5	1	143	127	30	0.8171	14	5	4	1	191	93	16	0.2970	14
Zeybek et al. [83] 2007	Turkish	Asia	Caucasian	52/144	HB	Non-gastric cancer	Age and sex	CRC	18	7	27	64	15	65	0	8	–	–	–	–	–	–	–	–
Osian et al. [84] 2007	Romania	European	Caucasian	69/67	HB	Non-gastric cancer	Age and sex	CRC	38	25	6	47	17	3	0.3779	12	33	32	4	41	25	1	0.1915	12
Guerreiro et al. [85] 2008	Portuguese	European	Caucasian	196/200	HB	Healthy controls	Age and sex	CRC	94	76	26	84	107	9	0.0005	12	–	–	–	–	–	–	–	–
Zhang, Y.L et al. [86] 2008	Liaoning	Asia	Asians	300/299, 299/300	HB	Non-gastric cancer	Age and sex	CRC	97	136	67	91	139	69	0.2598	15	189	93	17	196	89	15	0.2454	15
Eklöf et al. [87] 2008	Sweden	European	Caucasian	220/414	PB	Healthy controls	Age and sex	CRC	123	85	12	212	160	42	0.1527	16	–	–	–	–	–	–	–	–
Theodoratou et al. [88] 2008	Scotland	European	Caucasian	999/1010, 996/1009	PB	Healthy controls	Age and sex	CRC	447	441	111	439	455	116	0.9080	16	465	425	106	462	445	102	0.7333	16
Cao et al. [89] 2008	Jiangsu	Asia	Asians	315/371	HB	Healthy controls	Age and sex	CRC	109	154	52	121	183	66	0.8243	13	204	105	6	239	119	13	0.6998	13
Mokarram et al. [90] 2008	Iranian	Asia	Asians	151/81	HB	Healthy controls	Age and sex	colon	64	80	7	40	31	10	0.3097	12	–	–	–	–	–	–	–	–
Lightfoot et al. [91] 2008	UK	European	Caucasian	490/592, 488/593	HB	Non-gastric cancer	Age and sex	CRC	238	199	53	271	272	49	0.0918	14	231	200	57	288	259	46	0.2418	14
Kury et al. [92] 2008	French	European	Caucasian	1121/1023	PB	Healthy controls	Age and sex	CRC	457	515	149	435	452	136	0.2799	14	577	443	101	484	432	107	0.4679	14
Sharp et al. [93] 2008	Scotland	European	Caucasian	251/394, 245/394	PB	Healthy controls	Age and sex	CRC	117	111	23	170	177	47	0.9288	16	105	111	29	177	157	60	0.0124	14
Haghighi et al. [94] 2008	Iranian	Asia	Asians	234/257	HB	Non-gastric cancer	Age and sex	CRC	117	68	49	94	80	83	0	12	–	–	–	–	–	–	–	–
Haghighi et al. [95] 2009	Iranian	Asia	Asians	234/257	HB	Non-gastric cancer	Age and sex	CRC	117	68	49	76	118	63	0.2033	13	–	–	–	–	–	–	–	–
Derwinger et al. [96] 2009	Sweden	European	Caucasian	544/299	HB	Healthy controls	Age and sex	CRC	273	216	55	167	107	25	0.1897	15	–	–	–	–	–	–	–	–
El Awady et al. [97] 2009	Egypt	Asia	African	35/68	HB	Non-gastric cancer	NR	CRC	6	23	6	44	20	4	0.4071	8	5	21	9	26	37	5	0.0942	8
Reeves et al. [98] 2009	Poland	European	Caucasian	206/211	HB	Non-gastric cancer	Age and sex	CRC	105	83	18	101	91	19	0.8161	13	92	89	25	86	98	27	0.9109	13
Gallegos-Arreola et al. [99] 2009	Mexico	North American	Caucasian	369/170	PB	Healthy controls	Age and sex	CRC	124	126	119	59	79	32	0.5440	14	–	–	–	–	–	–	–	–
Iacopetta et al. [100] 2009	Australian	Oceania	mixed	850/958	PB	Non-gastric cancer	Age and sex	CRC	382	386	82	428	429	101	0.6710	16	–	–	–	–	–	–	–	–
de Vogel et al. [101] 2009	Netherlands	European	Caucasian	689/1793, 684/1767	PB	Non-gastric cancer	Age and sex	CRC	318	320	51	876	750	167	0.7234	16	299	275	110	735	774	258	0.0206	14
Zhu et al. [102] 2010	Jiangsu	Asia	Asians	216/111, 216/121	HB	Healthy controls	Age and sex	CRC	88	102	26	50	53	8	0.2276	13	90	102	24	50	61	10	0.1466	13
Fernandez-Peralta et al. [103] 2010	Spanish	European	Caucasian	143/103	HB	Healthy controls	Age and sex	CRC	89	52	2	44	50	9	0.3219	13	84	53	6	57	44	2	0.0476	11
Yang et al. [104] 2010	Jiangxi	Asia	Asians	141/165	HB	Healthy controls	Age and sex	CRC	58	61	22	62	75	28	0.5157	13	–	–	–	–	–	–	–	–
Promthet et al. [105] 2010	Thailand	Asia	Asians	130/130	HB	Non-gastric cancer	Age and sex	colon	104	26	0	94	31	5	0.2432	13	43	84	3	54	71	5	0.0018	11
Naghibalhossaini et al. [106] 2010	Iranian	Asia	Asians	151/231, 102/186	HB	Healthy controls	NR	CRC	64	80	7	150	68	13	0.1629	12	38	52	12	79	85	22	0.9055	12
Chandy et al. [107] 2010	India	Asia	Indian	100/86	HB	Non-gastric cancer	Age and sex	CRC	74	25	1	66	19	1	0.7767	11	22	70	8	22	50	14	0.1088	11
Eussen et al. [108] 2010	EPIC	European	Caucasian	1329/2366, 1330/2365	PB	Non-gastric cancer	Age and sex	CRC	567	608	154	1019	1076	271	0.6077	15	605	574	151	1099	1007	259	0.2154	15
Cui et al. [109] 2010	Korea	Asia	Asians	1829/1700	PB	Non-gastric cancer	Age	CRC	622	923	284	540	863	297	0.1326	14	–	–	–	–	–	–	–	–
Karpinski et al. [110] 2010	Poland	European	Caucasian	186/140	HB	Non-gastric cancer	Age and sex	CRC	74	97	15	71	55	14	0.4913	14	–	–	–	–	–	–	–	–
Komlosi et al. [111] 2010	Hungary	European	Caucasian	951/939	PB	Non-gastric cancer	Age and sex	CRC	398	427	126	442	380	117	0.0137	15	–	–	–	–	–	–	–	–
Wettergren et al. [112] 2010	Sweden	European	Caucasian	175/299	HB	Healthy controls	Age and sex	CRC	81	76	18	167	107	25	0.1897	13	–	–	–	–	–	–	–	–
Guimaraes et al. [113] 2011	Brazilian	South American	Caucasian	101/188	HB	Healthy controls	Age and sex	CRC	42	44	15	92	79	17	0.9945	13	62	33	6	127	49	12	0.0220	11
Guimaraes et al. [113] 2011	Brazilian	South American	African	12/188	HB	Healthy controls	Age and sex	CRC	6	6	0	92	79	17	0.9945	13	5	5	2	127	49	12	0.0220	11
Jokic et al. [114] 2011	Republic of Croatia	European	Caucasian	300/300	PB	Healthy controls	Age and sex	colon	139	130	31	142	130	28	0.8227	13	137	136	27	140	128	32	0.7341	13
Li et al. [115] 2011	China	Asia	Asians	137/145	HB	Non-gastric cancer	Age and sex	CRC	68	54	15	55	64	26	0.3326	13	88	47	2	76	60	9	0.5292	13
Prasad et al. [116] 2011	India	Asia	India	110/241	HB	NR	NR	CRC	97	12	1	228	12	1	0.0688	6	–	–	–	–	–	–	–	–
Kim et al. [117] 2011	Korea	Asia	Asians	67/53	HB	Non-gastric cancer	Age and sex	CRC	30	30	7	15	21	17	0.1329	11	44	22	1	36	16	1	0.6067	11
Pardini et al. [118] 2011	Czech	European	Caucasian	666/1376	HB	Non-gastric cancer	Age and sex	CRC	317	307	42	613	627	136	0.1840	16	281	309	76	583	638	156	0.3490	16
Zhu et al. [119] 2011	Jiangsu(China)	Asia	Asians	86/100	HB	Healthy controls	Age and sex	CRC	29	42	15	49	41	10	0.7421	11	–	–	–	–	–	–	–	–
Kang et al. [120] 2011	Korea	Asia	Asians	448/255	HB	Healthy controls	Age and sex	CRC	145	238	65	87	134	34	0.1160	14	–	–	–	–	–	–	–	–
Vossen et al. [121] 2011	Germany	European	Caucasian	1762/1811	HB	Non-gastric cancer	Age and sex	CRC	737	823	202	795	807	209	0.8463	14	–	–	–	–	–	–	–	–
Sameer et al. [122] 2011	India	Asia	India	86/160	HB	Non-gastric cancer	Age and sex	CRC	59	18	9	121	27	12	0	14	–	–	–	–	–	–	–	–
Yin et al. [123] 2012	Shandong	Asia	Asians	370/370	HB	Healthy controls	Age and sex	CRC	124	167	79	139	178	53	0.7420	11	–	–	–	–	–	–	–	–
Kim et al. [124] 2012	Korea	Asia	Asians	787/656	HB	Non-gastric cancer	Age and sex	CRC	265	393	129	205	289	162	0.0032	12	–	–	–	–	–	–	–	–
Promthet et al. [125] 2012	Thailand	Asia	Asians	112/242	HB	Non-gastric cancer	Age and sex	rectum	93	18	1	185	49	8	0.0444	11	37	74	1	85	147	10	0	11
Lee et al. [126] 2012 (a)	USA	North American	Caucasian	175/353, 175/355	PB	Healthy controls	Age	CRC	89	66	20	165	140	48	0.0407	13	72	82	21	181	136	38	0.1076	15
Lee et al. [126] 2012 (b)	USA	North American	Caucasian	158/318, 153/312	PB	Healthy controls	Age	CRC	72	69	17	140	127	51	0.0174	13	73	73	7	147	133	32	0.8129	15
Lee et al. [126] 2012 (c)	USA	North American	Caucasian	198/333, 213/365	PB	Healthy controls	Age	CRC	89	94	15	159	124	50	0.0025	13	101	100	12	167	154	44	0.3585	15
Sun et al. [127] 2012	Hebei	Asia	Asians	120/202	HB	Non-gastric cancer	Age and sex	CRC	54	56	10	66	96	40	0.6341	14	77	39	4	127	63	12	0.2730	14
Li et al. [128] 2012	Jiangxi	Asia	Asians	224/224	HB	Healthy controls	Age and sex	CRC	–	–	–	–	–	–	–	–	142	73	9	154	67	3	0.1488	14
Liu et al. [129] 2012	Jiangsu	Asia	Asians	52/52	HB	Non-gastric cancer	Age and sex	CRC	21	26	5	15	26	11	0.9658	10	–	–	–	–	–	–	–	–
Yousef et al. [130] 2013	jordan	Asia	Caucasian	128/106, 131/115	HB	Healthy controls	Age and sex	CRC	79	45	4	59	45	2	0.0453	10	51	59	21	43	58	14	0.4078	12
Delgado-Plasencia et al. [131] 2013	Spanish	European	Caucasian	50/103	HB	Healthy controls	NR	CRC	32	16	6	44	50	9	0.3219	9	–	–	–	–	–	–	–	–
Ashmore et al. [132] 2013	USA	North American	Caucasian	625/603	PB	Non-gastric cancer	Age and sex	CRC	241	309	75	263	259	81	0.1779	15	–	–	–	–	–	–	–	–
Ozen et al. [133] 2014	Turkish	Asia	Caucasian	86/212	HB	Healthy controls	NR	CRC	36	32	18	207	5	0	0.8621	10	27	47	12	209	3	0	0.9174	10
Rai et al. [134] 2014	India	Asia	India	155/294	HB	Non-gastric cancer	Age and sex	colon	137	17	1	261	31	2	0.3181	13	65	77	13	158	129	7	0.0010	11
Kim et al. [135] 2015	Korea	Asia	Asians	477/514	HB	Non-gastric cancer	Age and sex	CRC	159	248	70	172	265	77	0.1254	16	336	125	16	364	143	7	0.0890	16
Haerian et al. [17] 2016	Iranian	Asia	Caucasian	1123/1298	HB	Non-gastric cancer	Age and sex	CRC	607	421	95	667	523	108	0.7012	14	–	–	–	–	–	–	–	–
Zhang et al. [136] 2017	Fujian	Asia	Asians	383/1533	HB	Non-gastric cancer	Age and sex	CRC	177	175	31	639	697	197	0.7474	14	–	–	–	–	–	–	–	–
Shiao et al. [137] 2018	USA	North American	Caucasian	54/54	PB	Non-gastric cancer	Age and sex	CRC	23	25	6	28	21	5	0.7141	8	34	15	5	32	15	7	0.0314	6
Lin et al. [138] 2018	China	Asia	Asians	362/362	HB	Non-gastric cancer	Age and sex	CRC	232	108	22	185	134	43	0.0174	14	228	117	17	233	111	18	0.3176	16
Panprathip et al. [139] 2019	Thailand	Asia	Asians	105/182	HB	Healthy controls	Age and sex	CRC	73	31	1	140	39	3	0.8812	15	–	–	–	–	–	–	–	–
Mohd et al. [140] 2021	India	Asia	India	65/65	PB	Healthy controls	Age	CRC	41	18	6	47	15	3	0.2325	10	–	–	–	–	–	–	–	–

HB, hospital-based study; PB, population-based study; NR, not reported; NA, not available.

### Quality assessment

The study used the quality assessment scale based on the Preferred Reporting Entries for Systematic Reviews and Meta-Analyses (PRISMA) guidelines [141], the Guidelines for Reporting the Quality of Observational Studies [142,143], and the Quality Assessment of Prior Meta-Analyses [14]. Two authors independently assessed the quality of each included study. S1 Table provides information on the scores used to evaluate the quality of the studies included in the meta-analyses. The total quality scores ranged from 0-18. Articles with a score of 12 or more were considered high-quality, those with a score of 9-12 were considered moderate-quality, and those with a score less than 9 were considered low-quality.

### Statistical analysis

The study calculated the pooled odds ratios (*ORs*) and corresponding 95% confidence intervals (*95%CIs*) of gene frequencies for each of the five genetic models to assess the association between *MTHFR* polymorphisms and the risk of CRC. A statistically significant result was considered when *P* < 0.05. The five *MTHFR C677T* genetic models analysed were: (1) allele model T vs C; (2) additive model TT vs CC; (3) dominant model TT + CT vs CC; (4) recessive model TT vs CT + CC; and (5) super-dominant model CT vs CC. The *MTHFR A1298C* genetic models analysed were: (1) allele model C vs A; (2) additive model CC vs AA; (3) dominant model CC + AC vs AA; (4) recessive model CC vs AC + AA; and (5) super-dominant model AC vs AA.

Hardy-Weinberg equilibrium (*HWE*) was calculated for each study control group using a goodness-of-fit test. A significant disequilibrium (*HWD*) was defined as *P* < 0.05, otherwise, it was defined as *HWE* [144]. Subgroup analyses were conducted by stratifying studies based on the type of control matching, *HWE* condition, source of control, ethnicity, gender, tumour site, and TNM stage. Odds ratios (*ORs*) and corresponding 95% confidence intervals (*CIs*) were calculated to demonstrate the strength of this association.The meta-analysis’s heterogeneity was evaluated using the chi-square Q-test and *I*^*2*^ test. Results were interpreted as having no significant heterogeneity [145] if *P* > 0.10 and/or *I*^*2*^ ≤ 50%, and a fixed effects model (FEM) [146] was selected. If *P* < 0.10 and/or *I*^*2*^ > 50%, heterogeneity was considered large, and a random effects model (REM) [147] was chosen. To reduce heterogeneity, we used REM [147]. We also conducted subgroup analysis and meta-regression analysis to explore the source of heterogeneity. To assess the stability of the results, we performed sensitivity analyses using three methods: (1) excluding one study at a time; (2) excluding low- or medium-quality studies or those with *HWD*; and (3) retaining only high-quality and *HWE* studies. The study considered results to have no significant publication bias when Begg’s funnel plot [148] symmetry and Egger’s test suggested *P* > 0.05 [149]. In cases where significant publication bias was present, a nonparametric ‘trim and fill’ approach was used to correct and identify funnel plot asymmetries caused by publication bias while estimating the true value of the quantitative synthesis [150]. The False Positive Reporting Probability (FPRP) test [151] and the Venice Criterion [152] were applied to assess the credibility of statistically significant results. All statistical analyses were performed using STATA 12.0 (Stata Corp LP, College Station, Texas).

## Results

### Description of included studies

**Fig 1:**
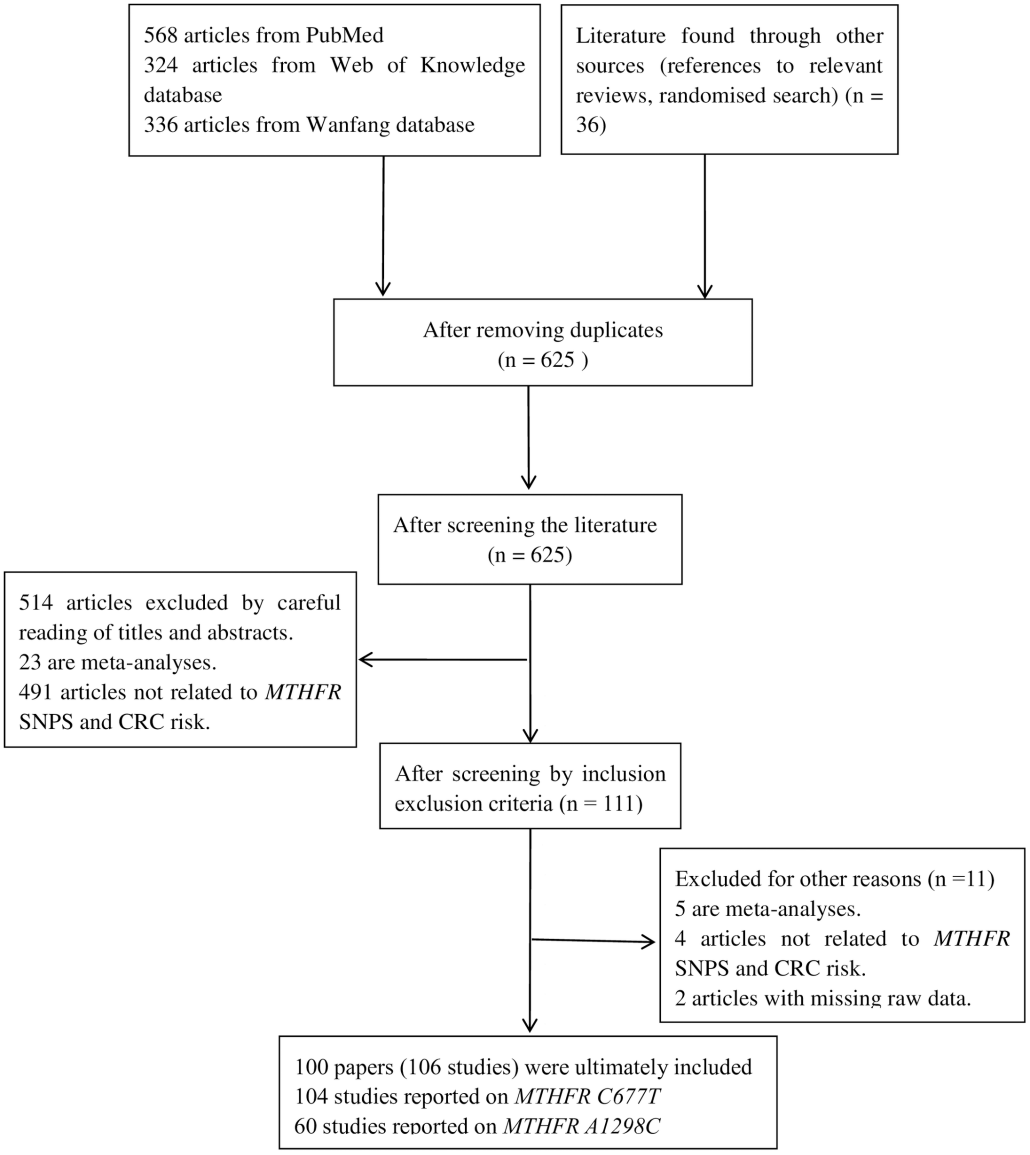
Flowchart of literature retrieval selection.

Following the established search strategy (refer to Fig 1 for detailed literature search and screening process), a total of 625 articles were obtained. After screening the titles, abstracts, and reading the full text in detail, 100 articles (106 studies) were selected for analysis. The publication year of the selected articles ranged from 1996-2023 [17,42–140]. Out of the selected studies, 104 described the association between the *MTHFR C677T* polymorphism and CRC susceptibility, while 60 studies described *MTHFR A1298C*.The analysis included 37 studies on Asians, 50 on Caucasians, 6 on Indians, 4 on Africans, and 7 on mixed-race populations. The quality of the studies was assessed, with 74 being of high quality (score >12) in describing the *MTHFR C677T* polymorphism and its association with CRC susceptibility. Additionally, there were 23 moderate-quality studies (scores between 9 and 12) and 7 low-quality studies (scores below 9). Regarding studies investigating the association between the *MTHFR A1298C* polymorphism and CRC risk, there were 43 high-quality studies, 15 moderate-quality studies, and 2 low-quality studies.The genotype distribution in the control groups of all studies, except for 17 [43,44,48,64,69,73,80,83,85,94,111,122,124–126,130,138], was consistent with the *HWE* test. Table 1 lists the genotype frequencies of the *MTHFR C677T* and *A1298C* polymorphisms associated with CRC risk, as well as the results of the *HWE* test and quality score.Additionally, we gathered genotype frequencies for confounding factors such as tumour type, location, gender, TNM stage, Duke stage, lymph node metastasis, degree of differentiation, smoking, and alcohol consumption. This was done by carefully reviewing the included literature to further investigate the role of these confounding factors in the pathogenic process of *MTHFR* gene polymorphisms in CRC. Subgroup analyses are detailed in Tables 2 and 3.

**Table 2:** Genotype frequencies of each genotype analyzed in the *MTHFR C677T* subgroup.

Categories	First author/Year	Variable	Genotypes distribution of *MTHFR C677T (rs1801133)*					
			Cases			Controls		
			CC	CT	TT	CC	CT	TT
Type of tumour	Matsuo et al. 2002	rectum	16	42	12	81	124	36
	**Jiang Q et al. 2004**	rectum	32	28	12	133	144	63
	Yin et al. 2004	rectum	110	144	36	278	367	133
	**Gao et al. 2005**	rectum	79	101	30	121	183	66
	Le Marchand et al.2005	rectum	99	90	31	987	779	255
	Liang et al.2006	rectum	33	15	8	90	50	3
	**Song et al. 2006**	rectum	44	51	16	174	174	61
	Wang et al. 2006	rectum	204	37	2	255	36	0
	Jin et al. 2007	rectum	99	117	35	211	325	136
	Murtaugh et al. 2007	rectum	357	301	84	466	392	112
	Cao et al.2008	rectum	79	101	30	121	183	66
	Komlosi et al. 2010	rectum	190	231	58	226	194	58
	Guimaraes et al. 2011	rectum	31	22	6	92	79	17
	Sameer et al. 2011	rectum	36	11	3	121	27	12
	Vossen et al. 2011	rectum	454	502	122	795	807	209
	Kim et al. 2012	rectum	109	164	57	205	289	162
	Promthet et al. 2012	rectum	93	18	1	185	49	8
	Sun QC **et al. 2013**	rectum	30	31	5	66	96	40
	Zhang et al. 2017	rectum	95	102	19	639	697	197
	Ghorbani et al. 2021	rectum	110	50	53	305	161	34
	Slattery et al. 1999	colon	673	655	139	827	787	207
	Slattery et al. 2000	colon	106	107	19	73	71	20
	Keku et al. 2002	colon	342	183	27	529	282	57
	Matsuo et al. 2002	colon	23	39	10	81	124	36
	Toffoli et al. 2003	colon	93	145	38	83	140	56
	Curtin et al. 2004	colon	734	724	150	887	858	227
	Jiang Q et al. 2004	colon	19	31	3	133	144	63
	Yin et al. 2004	colon	160	186	49	278	367	133
	Gao et al. 2005	colon	30	53	22	121	183	66
	Le Marchand et al. 2005	colon	295	246	56	987	779	255
	Song L et al. 2006	colon	31	51	11	174	174	61
	Wang et al. 2006	colon	53	6	0	255	36	0
	Jin et al. 2007	colon	82	92	20	211	325	136
	Mokarram et al. 2008	colon	64	80	7	40	31	10
	Cao et al. 2008	colon	30	53	22	121	183	66
	Promthet et al. 2010	colon	104	26	0	94	31	5
	Komlosi et al. 2010	colon	208	196	68	216	186	59
	Guimaraes et al. 2011	colon	17	28	9	92	79	17
	Sameer et al. 2011	colon	23	7	6	121	27	12
	Vossen et al. 2011	colon	283	321	80	795	807	209
	Jokic et al. 2011	colon	139	130	31	142	130	28
	Kim et al. 2012	colon	121	185	57	205	289	162
	Sun QC et al. 2013	colon	24	25	5	66	96	40
	Rai et al. 2014	colon	137	17	1	261	31	2
	Zhang et al. 2017	colon	82	73	12	639	697	197
	Ghorbani et al. 2021	colon	105	62	39	305	161	34
Gender	Slattery et al. 1999	male	372	378	74	435	423	109
		female	301	277	65	392	359	98
	Park et al. 1999	male	39	52	11	140	246	74
		female	26	55	17	140	246	74
	Shannon et al. 2002	male	115	96	20	533	560	114
		female	134	101	35	533	560	114
	Toffoli et al. 2003	male	51	75	24	51	88	34
		female	42	70	14	32	52	22
	Ulvik et al. 2004	male	710	560	102	695	562	133
		female	393	339	55	397	324	79
	Gao et al. 2005	male	67	95	28	72	118	33
		female	42	59	24	101	42	5
	Murtaugh et al. 2007	male	194	191	58	267	219	65
		female	163	110	26	199	173	47
	Cao et al. 2008	male	67	95	28	72	118	33
		female	42	59	24	49	65	33
	Mokarram et al. 2008	male	33	51	6	28	17	5
		female	31	29	1	12	14	5
	Lightfoot et al. 2008	male	115	116	22	147	150	25
		female	79	83	31	124	122	24
	Reeves et al. 2009	male	72	70	14	101	91	19
		female	134	104	23	101	91	19
	Iacopetta et al. 2009	male	206	174	37	240	260	62
		female	160	141	34	188	169	39
	de Vogel et al. 2009	male	179	184	19	409	405	87
		female	139	136	32	467	345	80
	Naghibalhossaini et al. 2010	male	33	51	6	87	46	7
		female	31	29	1	63	22	6
	Kang et al. 2011	male	46	81	16	87	134	34
		female	41	53	18	87	134	34
	Sameer et al. 2011	male	34	10	5	121	27	12
		female	25	8	4	121	27	12
	Zhu et al. 2011	male	24	10	16	49	41	10
		female	18	5	13	49	41	10
	Ghorbani et al. 2021	male	120	58	41	150	72	17
		female	95	54	51	155	89	17
Degree of differentiation/histopathology	Shannon et al. 2002	Well+moderate	210	174	47	533	560	114
		Poor	29	15	4	533	560	114
	Toffoli et al. 2003	Well+moderate	88	135	36	83	140	56
		Poor	5	10	2	83	140	56
	Lima et al. 2007	Well+moderate	33	41	15	143	127	30
		Poor	8	4	0	143	127	30
	Jin et al. 2007	Well+moderate	122	149	33	422	650	272
		Poor	60	62	23	211	325	136
	Naghibalhossaini et al. 2010	Well+moderate	58	66	7	150	68	13
		Poor	6	14	0	150	68	13
	Jin et al. 2007	Non-mucinous	157	187	42	422	650	272
		Mucinous	25	24	14	211	325	136
	Sameer et al. 2011	Non-mucinous	38	10	5	121	27	12
		Mucinous	21	8	4	121	27	12
	Sun QC et al. 2013	Non-mucinous	40	39	9	132	192	80
		Mucinous	14	17	1	66	96	40
Tumour position	Slattery et al. 1999	Proximal	339	319	64	827	787	207
		Distal	316	329	73	827	787	207
	Shannon et al. 2002	Proximal	129	118	31	533	560	114
		Distal	120	79	24	533	560	114
	Toffoli et al. 2003	Proximal	46	78	10	83	140	56
		Distal	47	67	28	83	140	56
	Yin et al. 2004	Proximal	59	75	16	278	367	133
		Distal	95	105	32	278	367	133
	Le Marchand et al. 2005	Proximal	202	200	53	987	779	255
		Distal	183	133	34	987	779	255
	Lima et al. 2007	Proximal	12	15	4	143	127	30
		Distal	28	29	11	143	127	30
	Mokarram et al. 2008	Proximal	22	37	1	40	31	10
		Distal	42	43	6	40	31	10
	Iacopetta et al. 2009	Proximal	107	137	31	188	169	39
		Distal	275	249	51	428	429	101
	Naghibalhossaini et al. 2010	Proximal	22	37	1	150	68	13
		Distal	42	43	6	150	68	13
Duke	Matsuo et al. 2002	A	2	14	1	439	326	83
		B	25	33	10	439	326	83
		A+B	27	47	11	878	652	166
		C	16	24	2	439	326	83
		D	3	2	1	439	326	83
		C+D	19	26	3	878	652	166
	Shannon et al. 2002	B	94	64	28	533	560	114
		C	155	133	27	533	560	114
	Toffoli et al. 2003	A+B	40	76	14	83	140	56
		C+D	53	69	24	83	140	56
	Osian et al. 2007	A	3	3	0	47	17	3
		B	16	12	2	47	17	3
		A+B	19	15	2	94	34	6
		C	15	7	1	47	17	3
		D	4	3	3	47	17	3
		C+D	19	10	4	94	34	6
	Jin et al. 2007	A	41	38	11	211	325	136
		B	62	74	20	211	325	136
		A+B	103	112	31	422	650	272
		C	54	64	14	211	325	136
		D	25	34	11	211	325	136
		C+D	79	98	25	422	650	272
	Sameer et al. 2011	A+B	26	8	4	121	27	12
		C+D	33	10	5	121	27	12
	Sun QC et al. 2013	A	9	10	1	66	96	40
		B	19	21	6	66	96	40
		A+B	28	31	7	132	192	80
		C	16	16	3	66	96	40
		D	10	9	0	66	96	40
		C+D	26	25	3	132	192	80
TNM	Park et al. 1999	I + II	18	36	10	140	246	74
		III + IV	32	46	11	140	246	74
	Lima et al. 2007	I + II	23	18	5	143	127	30
		III + IV	15	25	8	143	127	30
	Guimaraes et al. 2011	I + II	32	23	5	92	79	17
		III + IV	16	27	10	92	79	17
lymphatic node transfer	Jin et al. 2007	Not involved	114	123	37	211	325	136
		Involved	68	88	19	211	325	136
	Osian et al. 2007	Not involved	19	18	3	47	17	3
		Involved	19	7	3	47	17	3
	Sameer et al.2011	Involved	33	10	5	121	27	12
		Not involved	26	8	4	121	27	12
	Sun QC et al.2013	Not involved	30	29	7	66	96	40
		Involved	24	27	3	66	96	40
	Zhu et al.2011	Not involved	27	8	15	98	82	20
		Involved	15	10	11	98	82	20
Drinking	Jiang Q et al. 2004	NON-drinker	30	47	13	86	103	41
		Drinker	21	12	2	46	41	22
	Jin et al. 2007	NON-drinker	143	154	37	162	236	102
		Drinker	39	57	19	49	89	34
	Sun QC et al. 2013	NON-drinker	27	18	6	24	37	19
		Drinker	27	38	4	42	59	21
	Lin et al.2018	Drinkers	29	13	2	22	23	6
		Non-drinkers	203	95	20	163	111	37
Smoking	Naghibalhossaini et al. 2010	Never	40	44	3	23	24	9
		Current/past	24	36	4	17	7	1
	Sameer et al. 2011	Never	20	6	5	121	27	12
		Current/past	39	12	4	121	27	12
	Lin et al. 2018	Never	167	86	18	144	102	32
		Current/past	65	22	4	41	32	11

**Table 3:** Genotype frequencies of each genotype analyzed in the *MTHFR A1298C* subgroup.

Categories	First author/Year	Variable	Genotypes distribution of MTHFR A1298C (rs1801131)					
			Cases			Controls		
			AA	AC	CC	AA	AC	CC
Type of tumour	Matsuo et al. 2002	rectum	44	25	0	157	75	9
		colon	50	19	3	157	75	9
	Jiang Q et al. 2004	rectum	57	13	1	227	103	6
		colon	36	17	0	227	103	6
	Yin et al. 2004	rectum	192	90	8	515	244	19
		colon	246	130	19	515	244	19
	Gao et al. 2005	rectum	138	67	5	239	119	13
		colon	66	38	1	239	119	13
	He et al.2005	rectum	38	13	5	98	35	10
	Liang JZ et al. 2006	rectum	38	13	5	98	35	10
	Song L et al. 2006	rectum	75	32	1	265	140	6
		colon	58	30	1	265	140	6
	Wang et al. 2006	rectum	109	108	26	105	135	51
		colon	32	22	5	105	135	51
	Murtaugh et al. 2007	rectum	360	317	65	436	424	110
	Cao et al. 2008	rectum	138	67	5	239	119	13
		colon	66	38	1	239	119	13
	Guimaraes et al.2011	rectum	28	26	5	127	49	12
		colon	39	12	3	127	49	12
	Li FX et al. 2012	rectum	69	29	4	154	67	3
		colon	52	34	3	154	67	3
	Promthet et al. 2012	rectum	37	74	1	85	147	10
	Sun QC et al. 2013	rectum	44	20	2	127	63	12
		colon	33	19	2	127	63	12
Gender	Toffoli et al. 2003	male	70	65	15	87	71	15
		female	52	64	10	46	50	10
	Gao et al. 2005	male	124	62	4	138	77	8
		female	80	43	2	101	42	5
	Murtaugh et al. 2007	male	221	186	36	252	232	67
		female	139	131	29	184	192	43
	Cao et al. 2008	male	124	62	4	138	77	8
		female	80	43	2	101	42	5
	Lightfoot et al. 2008	male	125	133	38	158	150	15
		female	106	67	19	130	109	31
	Reeves et al. 2009	male	69	67	20	86	98	27
		female	109	120	32	86	98	27
	de Vogel et al. 2009	male	167	166	48	345	423	110
		female	132	109	62	390	351	148
	Naghibalhossaini et al. 2010	male	26	41	1	48	60	9
		female	12	11	11	31	25	13
	Li FX et al.2012	male	76	46	7	91	37	1
		female	66	27	6	63	30	2
Degree of differentiation/histopathology	Toffoli et al. 2003	Well+moderate	110	125	24	133	121	25
		Poor	12	4	1	133	121	25
	Lima et al. 2007	Well+moderate	60	24	5	191	93	16
		Poor	7	4	1	191	93	16
	Naghibalhossaini et al. 2010	Well+moderate	34	43	12	79	85	22
		Poor	0	5	0	79	85	22
	Guimaraes et al. 2011	Well+moderate	60	34	8	127	49	12
		Poor	7	4	0	127	49	12
	Sun QC et al.2013	Well+moderate	60	31	3	127	63	12
		Poor	17	8	1	127	63	12
Location	Toffoli et al. 2003	Proximal	57	70	7	133	121	25
		Distal	65	59	18	133	121	25
	Yin et al. 2004	Proximal	96	47	7	515	244	19
		Distal	140	81	11	515	244	19
	Lima et al. 2007	Proximal	23	7	1	191	93	16
		Distal	44	19	5	191	93	16
	Naghibalhossaini et al. 2010	Proximal	6	16	7	79	85	22
		Distal	32	36	5	79	85	22
Dukes	Toffoli et al. 2003	A+B	53	68	9	133	121	25
		C+D	69	61	16	133	121	25
	Osian et al. 2007	A+B	13	20	3	82	50	2
		C+D	20	12	1	82	50	2
	Sun QC et al. 2013	A+B	41	23	2	254	126	24
		C+D	36	16	2	254	126	24
Drinke	Jiang Q et al. 2004	NON-drinker	72	16	1	157	69	2
		Drinker	21	14	0	69	34	4
	Wang et al. 2006	NON-drinker	110	111	25	91	105	42
		Drinker	31	19	6	14	30	9
	Sun QC et al. 2013	NON-drinker	32	15	4	50	25	5
		Drinker	45	24	0	77	38	7

## Meta-analysis results

### Pooled analysis for *MTHFR* C677T (*rs1801133*)

**Fig 2:**
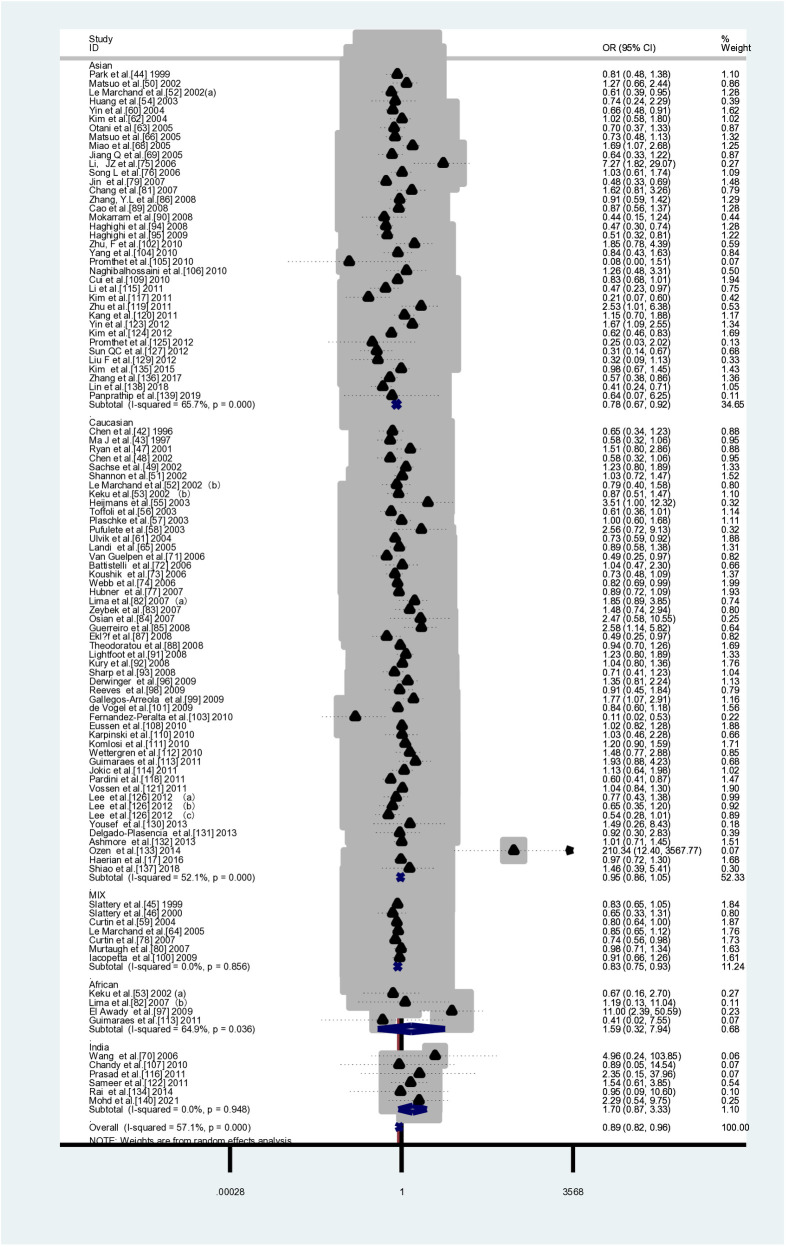
Forest plot of ethnic subgroup analyses of the *MTHFR C677T* polymorphism in association with CRC risk. TT vs. CC (overall data).

This meta-analysis included 104 case-control studies (41,884 cases and 58,362 controls) that investigated the association between *MTHFR C677T* and CRC susceptibility. Table 4 shows that two genetic models demonstrated a decreased risk of colorectal cancer due to *MTHFR* when compared to controls: TT vs CC (*OR*=0.888, 95% *CI*=0.822-0.959) and TT vs CC+CT (*OR*=0.891, 95% *CI*=0.831-0.956).In the subgroup analyses based on ethnicity, it was observed that the *MTHFR C677T* gene polymorphism was found to be a susceptibility factor for CRC in Indian ethnic groups(CT+TT vs CC: *OR*=1.329, *95%CI*=1.033-1.709 (FEM); T vs C: *OR*=1.336,*95%CI*=1.065-1.675 (FEM)), while significantly reducing the risk of CRC in Asian and mixed races(Asian TT vs CC: *OR*=0.783, *95%CI*=0.669-0.917; TT vs CC+CT: *OR*=0.811, *95%CI*=0.708-0.929; T vs C: *OR*=0.906, *95%CI*=0.836-0.981; mixed race TT vs CC: *OR*=0.831, *95%CI*=0.746-0.926 (FEM); TT vs CC+CT: *OR*=0.824, *95%CI*=0.743-0.914 (FEM); T vs C: *OR*=0.946, *95%CI*=0.902- 0.993 (FEM)).It has been observed that there are negative correlations between *MTHFR* gene polymorphisms and susceptibility to CRC in both population-based control groups and control groups from non-cancer studies.(PB：TT vs CC: *OR*=0.850, *95%CI*=0.804-0.898 (FEM); TT vs CC+CT: *OR*=0.845, *95%CI*=0.802-0.890 (FEM); T vs C: *OR*=0.954, *95%CI*= 0.931-0.978 (FEM);Non-cancer controls:TT vs CC: *OR*=0.834, *95%CI*=0.792-0.877 (FEM); TT vs CC+CT: *OR*=0.834, *95%CI*=0.795-0.875 (FEM)). Table 4 and Fig 2 show the results of the pooled analyses and racial distribution.

**Table 4:** Meta-analysis of the association of *MTHFR C677T* polymorphism with risk of CRC.

Variable		n (Cases/Controls)	TT vs. CC		CT vs.CC		TT vs. CC+CT		CT+TT vs. CC		T vs. C	
			*OR (95% CI)*	*Ph/I ^*2*^ *(%)*	*OR (95% CI)*	*Ph/I ^*2*^ *(%)*	*OR (95% CI)*	*Ph/I ^*2*^ *(%)*	*OR (95% CI)*	*Ph/I ^*2*^ *(%)*	*OR (95% CI)*	*Ph/I^*2*^ *(%)*
**Overall**	REM	104 (41884/58362)	**0.888 (0.822-0.959)**	**0.000/57.1**	1.010(0.962-1.061)	0.000/57.5	**0.891(0.831-0.956)**	**0.000/54.2**	0.988(0.939-1.039)	0.000/64.1	0.971(0.933-1.011)	0.000/69.4
**Ethnicity**												
Asian	REM	37(10470/13621)	**0.783(0.669-0.917)**	**0.000/65.7**	0.957(0.871-1.052)	0.000/55.5	**0.811(0.708-0.929)**	**0.000/61.7**	0.916(0.829-1.013)	0.000/65.2	**0.906(0.836-0.981)**	**0.000/72.5**
Caucasian	REM	50 (23663/32841)	0.953(0.864-1.051)	0.000/52.1	1.021(0.953-1.095)	0.000/63.4	0.950(0.863-1.046)	0.000/55.5	1.013(0.946-1.084)	0.000/66.2	0.999(0.947-1.053)	0.000/69.0
India	REM	6 (818/1137)	1.699(0.866-3.335)	0.948/0.0	1.288(0.989-1.677)	0.751/0.0	1.598(0.820-3.116)	0.951/0.0	**1.334(1.037-1.716)**	**0.703/0.0**	**1.340(1.068-1.681)**	**0.658/0.0**
	FEM		1.739(0.899-3.366)	0.948/0.0	1.283(0.985-1.670)	0.751/0.0	1.637(0.851-3.148)	0.951/0.0	**1.329(1.033-1.709)**	**0.703/0.0**	**1.336(1.065-1.675)**	**0.658/0.0**
MIX	REM	7(6632/9878)	**0.831(0.746-0.927)**	**0.856/0.0**	1.017(0.951-1.087)	0.987/0.0	**0.825(0.744-0.914)**	**0.845/0.0**	0.977(0.917-1.041)	0.983/0.0	**0.946(0.902-0.993)**	**0.944/0.0**
	FEM		**0.831(0.746-0.926)**	**0.856/0.0**	1.017(0.951-1.087)	0.987/0.0	**0.824(0.743-0.914)**	**0.845/0.0**	0.977(0.917-1.041)	0.983/0.0	**0.946(0.902-0.993)**	**0.944/0.0**
African	REM	4(301/885)	1.593(0.320-7.937)	0.036/64.9	1.864(0.671-5.178)	0.003/78.8	1.241(0.490-3.140)	0.322/14.0	1.789(0.613-5.223)	0.001/81.8	1.374(0.632-2.986)	0.001/81.0
	FEM						1.198(0.550-2.612)	0.322/14.0				
**Source of control**												
HB	REM	64(16060/22486)	0.959(0.833-1.104)	0.000/66.1	1.016(0.925-1.116)	0.000/70.2	0.966(0.854-1.091)	0.000/61.1	1.015(0.922-1.118)	0.000/75.3	1.008(0.935-1.088)	0.000/78.3
PB	REM	40(25824/35876)	**0.849(0.792-0.909)**	**0.077/25.3**	1.022(0.987-1.058)	0.888/0.0	**0.841(0.784-0.902)**	**0.024/33.0**	0.984(0.952-1.017)	0.797/0.0	**0.954(0.927-0.982)**	**0.180/16.9**
	FEM		**0.850(0.804-0.898)**	**0.077/25.3**	1.022(0.987-1.058)	0.888/0.0	**0.845(0.802-0.890)**	**0.024/33.0**	0.984(0.952-1.017)	0.797/0.0	**0.954(0.931-0.978)**	**0.180/16.9**
**Type of control**												
Healthy	REM	39(9775/12835)	1.057(0.894-1.249)	0.000/63.4	1.049(0.930-1.183)	0.000/72.1	1.059(0.906-1.237)	0.000/63.3	1.069(0.947-1.206)	0.000/75.4	1.064(0.969-1.168)	0.000/77.9
Non-cancer	REM	62(29822/42303)	**0.820(0.756-0.890)**	**0.000/47.4**	0.996(0.949-1.045)	0.001/40.4	**0.827(0.772-0.885)**	**0.003/36.4**	0.954(0.907-1.003)	0.000/51.0	0.929(0.892-0.968)	0.000/58.4
	FEM		**0.834(0.792-0.877)**	**0.000/47.4**	1.002(0.970-1.035)	0.001/40.4	**0.834(0.795-0.875)**	**0.003/36.4**				
**HWE and Quality score > 12**												
**Overall**	REM	63 (29156/39419)	**0.876(0.806-0.952)**	**0.000/48.6**	0.985(0.944-1.029)	0.030/26.6	**0.891(0.827-0.960)**	**0.000/44.0**	0.961(0.917-1.006)	0.001/39.8	**0.953(0.917-0.990)**	**0.000/52.1**
	FEM		**0.866(0.822-0.913)**	**0.000/48.6**	0.991(0.958-1.025)	0.030/26.6	**0.879(0.837-0.922)**	**0.000/44.0**	**0.966(0.935-0.997)**	**0.001/39.8**		
**Ethnicity**												
Asian	REM	23(7635/10193)	**0.804(0.683-0.945)**	**0.000/57.9**	**0.922(0.838-1.015)**	**0.019/42.0**	**0.849(0.745-0.969)**	**0.007/47.4**	**0.893(0.805-0.990)**	**0.001/55.0**	**0.905(0.836-0.981)**	**0.000/63.3**
	FEM				**0.922(0.861-0.986)**	**0.019/42.0**	**0.836(0.768-0.911)**	**0.007/47.4**				
Caucasian	REM	30(15725/20937)	0.953(0.851-1.068)	0.002/47.4	1013(0.955-1.073)	0.090/26.8	0.952(0.851-1.064)	0.001/51.1	1.000(0.944-1.060)	0.048/32.2	0.989(0.941-1.039)	0.002/47.5
	FEM		0.938(0.873-1.009)	0.002/47.4	1.016(0.971-1.063)	0.090/26.8			1.001(0.959-1.045)	0.048/32.2	0.985(0.954-1.017)	0.002/47.5
MIX	REM	5(5073/6887)	**0.805(0.708-0.914)**	**0.866/0.0**	1.006(0.932-1.087)	0.983/0.0	**0.802(0.710-0.906)**	**0.867/0.0**	0.965(0.896-1.038)	0.966/0.0	**0.935(0.885-0.988)**	**0.914/0.0**
	FEM		**0.804(0.708-0.913)**	**0.866/0.0**	1.006(0.932-1.087)	0.983/0.0	**0.802(0.710-0.905)**	**0.867/0.0**	0.965(0.896-1.038)	0.966/0.0	**0.935(0.885-0.988)**	**0.914/0.0**
African	REM	3(266/817)	0.716(0.239-2.145)	0.836/0.0	1.023(0.693-1.510)	0.852/0.0	0.690(0.235-2.028)	0.872/0.0	0.976(0.669-1.424)	0.866/0.0	0.933(0.669-1.301)	0.816/0.0
	FEM		0.691(0.232-2.061)	0.836/0.0	1.023(0.694-1.509)	0.852/0.0	0.667(0.228-1.955)	0.872/0.0	0.976(0.670-1.424)	0.866/0.0	0.931(0.668-1.299)	0.816/0.0
**Source of control**												
HB	REM	37(11214/24156)	0.902(0.777-1.048)	0.000/58.0	0.957(0.882-1.038)	0.003/43.6	0.930(0.822-1.052)	0.001/47.9	0.946(0.867-1.003)	0.000/55.9	0.955(0.890-1.024)	0.000/63.3
	FEM				0.962(0.910-1.016)	0.003/43.6	**0.912(0.843-0.986)**	**0.001/47.9**				
PB	REM	26(17942/30549)	0.858(0.789-0.934)	0.104/26.8	1.009(0.967-1.052)	0.802/0.0	**0.860(0.788-0.938)**	**0.031/37.0**	0.976(0.938-1.016)	0.718/0.0	**0.954(0.921-0.988)**	**0.178/20.2**
	FEM		**0.856(0.801-0.914)**	**0.104/26.8**	1.009(0.967-1.052)	0.802/0.0	**0.858(0.807-0.914)**	**0.031/37.0**	0.976(0.938-1.016)	0.718/0.0	**0.953(0.925-0.982)**	**0.178/20.2**
**Type of control**												
Healthy	REM	22(6971/7605)	0.981(0.809-1.190)	0.000/61.4	0.976(0.898-1.062)	0.218/18.3	1.009(0.850-1.199)	0.000/58.5	0.980(0.887-1.083)	0.011/45.7	0.996(0.910-1.091)	0.000/65.3
	FEM				0.979(0.911-1052)	0.218/18.3			0.979(0.915-1.049)	0.011/45.7		
Non-cancer	REM	40(22167/31021)	0.832(0.767-0.901)	0.036/30.8	0.988(0.938-1.040)	0.023/33.4	**0.844(0.789-0.903)**	**0.162/18.1**	0.953(0.905-1.003)	0.009/38.2	0.936(0.901-0.972)	0.009/38.2
	FEM		**0.837(0.788-0.888)**	**0.036/30.8**	0.994(0.957-1.032)	0.023/33.4	**0.844(0.798-0.893)**	**0.162/18.1**	**0.961(0.927-0.996)**	**0.009/38.2**	0.936(0.901-0.972)	0.009/38.2
**Egger’s test**												
***P***_***E***_			0.192		**0.008**		0.548		0.011		0.031	

HB, hospital-based studies; PB, population-based studies; REM, Random effects model; FEM, Fixed effects model. *P<0.05 was considered statistically significant.

Table 5 presents the details of an extensive subgroup analysis that was conducted to further investigate the association between the *MTHFR C677T* polymorphism and susceptibility to CRC. In subgroup analyses based on tumour site, it was observed that *MTHFR* gene polymorphisms were associated with a reduced risk of colon cancer only in the TT vs. CC+CT model (*OR*=0.833, *95%CI*=0.698-0.994). But no significant association was found between *MTHFR* gene polymorphisms and rectal cancer susceptibility. The results of the stratified analysis indicate that a low degree of differentiation is significantly associated with a reduced risk of CRC (TT vs CC: *OR*=0.592, *95%CI*=0.380-0.923 (FEM)). However, no association was found in all genetic models for the highly and moderately differentiated studies. On the other hand, the genetic models for non-mucinous carcinoma studies suggest that *MTHFR* polymorphisms may act as protective factors(TT vs CC: *OR*=0.503, *95%CI*=0.281-0.899; CT vs CC: *OR*=0.775,*95%CI=*0.627-0.958 (FEM); TT vs CC+CT: *OR*=0.509, *95%CI*=0.377-0.687 (FEM); CT+TT vs CC: *OR*=0.685, *95%CI*=0.561-0.838 (FEM); T vs C. *OR*=0.729,*95%CI*=0.552-0.964). In contrast, there is no association between mucinous colorectal cancer susceptibility and *MTHFR* polymorphisms. Of note, in the subgroup analysis based on tumour location, it was observed that *MTHFR* polymorphisms were identified as a risk factor for proximal CRC (CT vs CC: *OR*=1.251,*95%CI*=1.003-1.560) and a protective factor for distal CRC(TT vs CC: *OR*=0.853, *95%CI*=0.729-0.997 (FEM)). In the analysis stratified by Dukes staging, it was observed that *MTHFR* reduced susceptibility to stage A and C CRC (stage A CRC:TT vs CC: *OR*=0.434, *95%CI*=0.234-0.808 (FEM); TT vs CC+CT: *OR*=0.505, *95%CI*=0.282-0.907 (FEM);stage C CRC:TT vs CC: *OR*=0.601, *95%CI*=0.428-0.844 (FEM); TT vs CC+CT: *OR*=0.646, *95%CI*=0.468-0.892 (FEM); T vs C: *OR*=0.821,*95%CI*=0.712-0.945 (FEM)), but not in relation to Stage B and D CRC. Similar results were observed in both drinking and non-drinking studies when analyses were stratified according to alcohol consumption. Additionally, subgroup analyses were performed according to gender, TNM stage and lymph node metastasis, and smoking status, and no associations were observed.

**Table 5:** Stratified analysis of association studies between polymorphisms at the *MTHFR C677T* locus and risk of developing CRC.

Variable		TT vs.CC		CT vs.CC		TT vs. CC+CT		CT+TTvs.CC		T vs. C	
		*OR (95% CI)*	*Ph/I2 (%)	*OR (95% CI)*	*Ph/I2 (%)	*OR (95% CI)*	*Ph/I2 (%)	*OR (95% CI)*	*Ph/I2 (%)	*OR (95% CI)*	*Ph/I2 (%)
**Subgroupanalysis**											
**Location**											
Rectum 20 (5078/12375)	REM	0.961 (0.746-1.238)	0.000/75.1	1.018 (0.938-1.105)	0.328/10.2	0.957 (0.755-1.212)	0.000/75.2	0.999 (0.901-1.108)	0.019/43.8	0.988 (0.887-1.101)	0.000/72.4
	FEM			1.024 (0.950-1.103)	0.328/10.2			1.014 (0.945-1.088)	0.019/43.8		
Colon 26 (8580/16912)	REM	0.857 (0.706-1.040)	0.000/69.8	1.033 (0.974-1.095)	0.590/0.0	**0.833 (0.698-0.994)**	**0.000/68.1**	0.995 (0.917-1.079)	0.023/38.9	0.959 (0.884-1.041)	0.000/66.3
	FEM			1.033 (0.974-1.095)	0.590/0.0			0.990 (0.937-1.047)	0.023/38.9		
**Gendering**											
Male 18 (5351/8134)	REM	0.963 (0.781-1.187)	0.002/56.9	1.008 (0.895- 1.136)	0.009/49.7	0.971 (0.794-1.188)	0.001/58.2	1.015 (0.902-1.141)	0.004/53.3	1.016 (0.922- 1.119)	0.001/62.2
	FEM			0.996 (0.923- 1.074)	0.009/49.7						
Female 18 (4076/6803)	REM	1.230 (0.895-1.690)	0.000/77.8	1.056 (0.902- 1.236)	0.000/62.9	1.219 (0.899-1.652)	0.000/78.3	1.114 (0.946-1.312)	0.000/70.2	1.130 (0.972-1.313)	0.000/80.7
**Differentiation**											
Well + moderate 5 (1214/3361)	REM	0.899 (0.517-1.565)	0.000/80.9	1.107 (0.758-1.615)	0.005/83.3	0.878 (0.543-1.420)	0.001/77.8	1.072 (0.727- 1.580)	0.000/85.7	1.024 (0.763-1.373)	0.000/86.9
Poor 5 (242/2689)	REM	**0.597 (0.383-0.931)**	**0.986/0.0**	0.972 (0.471-2.007)	0.002/76.7	0.722 (0.478-1.090)	0.950/0.0	0.878 (0.458- 1.684)	0.005/73.3	0.839 (0.566-1.244)	0.043/59.3
	FEM	**0.592 (0.380-0.923)**	**0.986/0.0**			0.711 (0.471-1.073)	0.950/0.0				
Mucinous 3 (128/1034)	REM	0.759 (0.233-2.470)	0.063/63.8	0.883 (0.508-1.533)	0.192/39.5	0.850 (0.281-2.575)	0.072/62.1	0.882 (0.488- 1.595)	0.107/55.2	0.915 (0.529-1.584)	0.035/70.1
	FEM			0.829 (0.545-1.260)	0.192/39.5						
Non-mucinous 3 (527/1636)	REM	**0.503 (0.281-0.899)**	**0.129/51.2**	0.775 (0.627-0.958)	0.507/0.0	**0.546 (0.350-0.852)**	**0.231/31.7**	**0.706 (0.520- 0.958)**	**0.205/36.9**	**0.729 (0.552-0.964)**	**0.116/53.6**
	FEM			**0.775 (0.627-0.958)**	**0.507/0.0**	**0.509 (0.377-0.687)**	**0.231/31.7**	**0.685 (0.561- 0.838)**	**0.205/36.9**		
**Location**											
Proximal 9 (2165/7114)	REM	0.828 (0.613-1.120)	0.022/55.4	**1.251 (1.003-1.560)**	**0.000/71.3**	0.777 (0.578-1.046)	0.014/58.4	1.159 (0.944-1.422)	0.001/69.6	1.021 (0.885-1.177)	0.003/65.9
Distal 9 (2490/7676)	REM	0.858 (0.734-1.004)	0.449/0.0	0.982 (0.816-1.181)	0.004/65.0	0.886 (0.764-1.028)	0.504/0.0	0.963 (0.813-1.141)	0.006/62.9	0.955 (0.854-1.067)	0.033/52.3
	FEM	**0.853 (0.729-0.997)**	**0.449/0.0**			0.880 (0.759-1.020)	0.504/0.0				
**Dukes**											
A 4 (133/1789)	REM	0.517 (0.209-1.279)	0.298/18.4	1.554 (0.499-4.842)	0.002/79.2	**0.527 (0.293-0.949)**	**0.754/0.0**	1.304 (0.431-3.944)	0.003/79.0	0.967 (0.488-1.918)	0.006/75.8
	FEM	**0.434 (0.234-0.808)**	**0.298/18.4**	0.949 (0.652-1.380)	0.002/79.2	**0.505 (0.282-0.907)**	**0.754/0.0**				
B 5 (486/2996)	REM	1.015 (0.535-1.927)	0.007/71.3	0.989 (0.646-1.514)	0.009/70.3	1.048 (0.591-1.859)	0.012/68.7	0.999 (0.660-1.511)	0.006/72.3	1.018 (0.740-1.399)	0.003/75.0
C 5 (547/2996)	REM	**0.595 (0.400-0.883)**	**0.339/11.7**	0.947 (0.678-1.322)	0.093/49.8	**0.651 (0.452-0.937)**	**0.356/8.9**	0.863 (0.623-1.195)	0.085/51.2	0.827 (0.658-1.040)	0.118/45.6
	FEM	**0.601 (0.428-0.844)**	**0.339/11.7**	0.881 (0.725-1.071)	0.093/49.8	**0.646 (0.468-0.892)**	**0.356/8.9**			**0.821 (0.712-0.945)**	**0.118/45.6**
D 4 (105/1789)	REM	1.168 (0.206-6.615)	0.011/73.1	0.874 (0.563-1.357)	0.652/0.0	1.227 (0.259-5.805)	0.019/70.0	0.936 (0.455-1.927)	0.105/51.1	1.056 (0.471-2.363)	0.004/77.3
	FEM			0.872 (0.562-1.353)	0.652/0.0						
**TNM**											
I,II 3 (170/1408)	REM	0.990 (0.573-1.709)	0.946/0.0	0.953 (0.667-1.361)	0.748/0.0	0.985 (0.596-1.626)	0.967/0.0	0.954 (0.680-1.339)	0.773/0.0	0.973 (0.763-1.242)	0.861/0.0
	FEM	0.988 (0.573-1.706)	0.946/0.0	0.953 (0.669-1.359)	0.748/0.0	0.984 (0.596-1.625)	0.967/0.0	0.955 (0.682-1.338)	0.773/0.0	0.973 (0.763-1.241)	0.861/0.0
III,IV 3 (190/1408)	REM	1.709 (0.580-5.039)	0.009/78.8	1.383 (0.748-2.559)	0.048/67.2	1.397 (0.667-2.922)	0.068/62.7	1.464 (0.714-3.002)	0.010/78.1	1.335 (0.778-2.291)	0.004/82.0
	FEM			1.252 (0.894-1.755)	0.048/67.2						
**Lymph nodes**											
Involved 5 (342/1301)	REM	1.005 (0.342-2.950)	0.000/82.5	0.878 (0.672-1.147)	0.840/0.0	1.067 (0.366-3.105)	0.000/83.8	0.913 (0.649-1.285)	0.184/35.6	1.025 (0.655-1.604)	0.001/78.1
	FEM			0.877 (0.670-1.146)	0.840/0.0			0.855 (0.666-1.097)	0.184/35.6		
Not involved 5 (468/1301)	REM	1.046 (0.444-2.461)	0.001/78.8	0.861 (0.507-1.463)	0.009/70.3	1.174 (0.497-2.774)	0.000/81.0	0.941 (0.588-1.507)	0.009/70.5	1.057 (0.692-1.613)	0.001/79.5
**Drink**											
NON-drinker 4(793/1121)	REM	**0.469 (0.319-0.690)**	**0.233/30.0**	0.760 (0.553-1.046)	0.102/51.7	**0.526 (0.395-0.698)**	**0.652/0.0**	**0.675 (0.485-0.939)**	**0.058/59.8**	**0.823 (0.731-0.927)**	**0.540/0.0**
	FEM	**0.459 (0.339-0.622)**	**0.233/30.0**			**0.524 (0.395-0.697)**	**0.652/0.0**			**0.823 (0.731-0.926)**	**0.540/0.0**
Drinker 4 (263/454)	REM	**0.422 (0.227-0.785)**	**0.309/16.6**	0.751 (0.536-1.053)	0.463/0.0	**0.474 (0.250-0.902)**	**0.248/27.4**	**0.656 (0.476-0.906)**	**0.401/0.0**	**0.813 (0.666-0.992)**	**0.825/0.0**
	FEM	**0.432 (0.258-0.725)**	**0.309/16.6**	0.750 (0.536-1.049)	0.463/0.0	**0.511 (0.319-0.821)**	**0.248/27.4**	**0.655 (0.476-0.901)**	**0.401/0.0**	**0.812 (0.666-0.991)**	**0.825/0.0**
**Smoking**											
Non-smokers 3 (1355/1839)	REM	0.633 (0.178-2.252)	0.011/77.9	0.826 (0.607-1.124)	0.401/0.0	0.644 (0.192-2.163)	0.013/77.0	0.882 (0.534-1.454)	0.116/53.6	0.895 (0.742-1.081)	0.345/6.1
	FEM			0.824 (0.605-1.122)	0.401/0.0					0.892 (0.748-1.065)	0.345/6.1
Smokers 3 (1176/1614)	REM	0.715 (0.186-2.743)	0.081/60.1	1.235 (0.379-4.024)	0.002/84.4	0.642 (0.254-1.619)	0.271/23.4	1.144 (0.341-3.842)	0.000/87.3	0.944 (0.710-1.255)	0.267/24.4
	FEM			0.996 (0.645-1.540)	0.002/84.4	0.612 (0.287-1.303)	0.271/23.4			0.934 (0.733-1.190)	0.267/24.4

HB, hospital-based studies; PB, population-based studies; REM, Random effects model; FEM, Fixed effects model. *P<0.05 was considered statistically significant.

### Pooled analysis for *MTHFR A1298C* (*rs1801131*)

**Fig 3:**
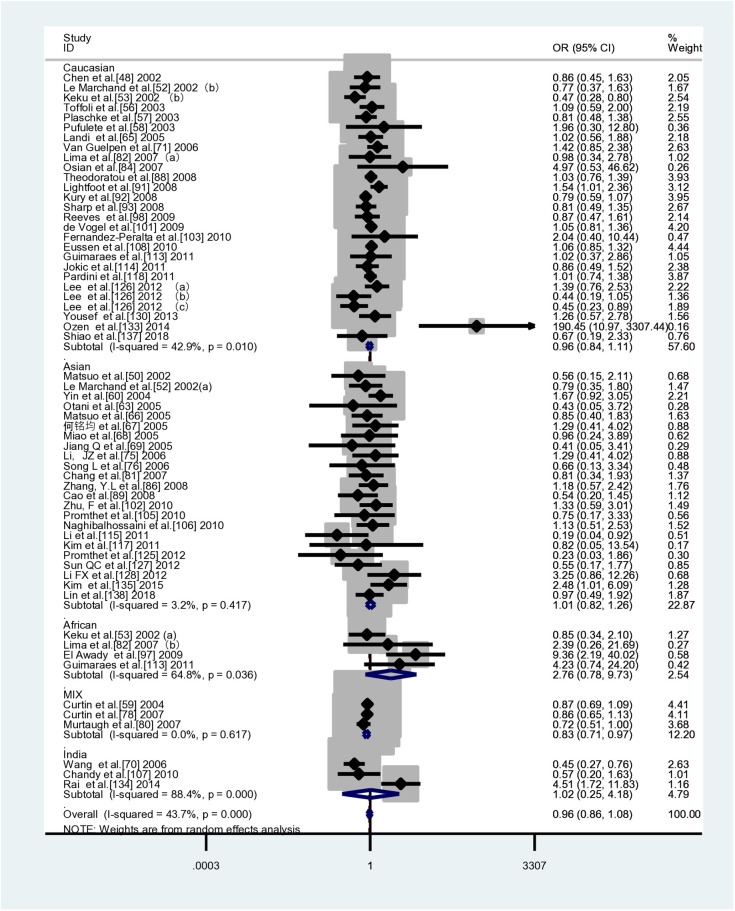
Forest plot of ethnic subgroup analyses of the *MTHFR A1298C* polymorphism in correlation with CRC risk. CC vs. AA (overall data).

Table 6 and Fig 3 present the results of pooled analyses of the MTHFR A1298C polymorphism and CRC susceptibility, including ethnic distribution. In the meta-analysis, a total of 60 case-control studies were included, comprising of 18,103 cases and 26,970 controls. It was observed that none of the genetic models showed any statistically significant associations. The subgroup analyses based on ethnicity and type of control also yielded similar results. Nevertheless, in population-based control studies, three genetic models suggest that the *MTHFR C677T* polymorphism is associated with a reduced risk of CRC (CC vs AA: *OR*=0.895,*95% CI*=0.822-0.976 (FEM); CC vs AA+AC: *OR*=0.900,*95% CI*=0.829-0.977 (FEM); C vs A: *OR* =0.961, 95% *CI*=0.926-0.998 (FEM)).

**Table 6:** Meta-analysis of the association of *MTHFR A1298C* polymorphism with risk of CRC.

Variable		n (Cases/Controls)	CC vs.AA		CA vs. AA		CC vs. AA+AC		AC +CC vs.AA		C vs. A	
			*OR (95% CI)*	*Ph/I2(%)	*OR (95% CI)*	*Ph/I2(%)	*OR (95% CI)*	*Ph/I2(%)	*OR (95% CI)*	*Ph/I2(%)	*OR (95% CI)*	*Ph/I2(%)
**Overall**	REM	60 (18103/26970)	0.962 (0.856-1.081)	0.000/43.7	1.025 (0.962-1.092)	0.000/46.4	0.949 (0.855-1.053)	0.003/36.3	1.018 (0.953-1.087)	0.000/54.7	1.002 (0.949-1.057)	0.000/58.7
	FEM		0.952 (0.886-1.023)	0.000/43.7	1.015 (0.974-1.057)	0.000/46.4	0.949 (0.885-1.016)	0.003/36.3				
**Ethnicity**												
Asian	REM	23 (4891/6902)	1.015 (0.818-1.258)	0.417/3.2	0.995 (0.917-1.080)	0.847/0.0	1.003 (0.812-1.239)	0.422/2.8	0.996 (0.921-1.078)	0.739/0.0	0.997 (0.933-1.065)	0.630/0.0
	FEM		0.998 (0.815-1.222)	0.417/3.2	0.994 (0.917-1.079)	0.847/0.0	0.986 (0.808-1.204)	0.422/2.8	0.995 (0.920-1.077)	0.739/0.0	0.995 (0.931-1.063)	0.630/0.0
Caucasian	REM	27 (9089/13598)	0.963 (0.836-1.110)	0.010/42.9	1.055 (0.943-1.181)	0.000/67.3	0.961 (0.846-1.091)	0.033/36.1	1.044 (0.933-1.168)	0.000/70.6	1.014 (0.931-1.104)	0.000/70.5
	FEM		0.989 (0.902-1.085)	0.010/42.9			0.990 (0.907-1.081)	0.033/36.1				
African	REM	4 (300/885)	2.758 (0.782-9.725)	0.036/64.8	1.547 (0.911-2.628)	0.231/30.2	1.953 (0.790-4.829)	0.147/44.1	1.859 (0.930-3.717)	0.071/57.4	1.663 (0.969-2.852)	0.030/66.4
	FEM				1.292 (0.940-1.776)	0.231/30.2	1.643 (0.894-3.021)	0.147/44.1				
India	REM	3 (557/671)	1.024 (0.251-4.182)	0.000/88.4	1.097 (0.653-1.842)	0.022/73.8	0.935 (0.285-3.065)	0.001/86.0	1.064 (0.559-2.025)	0.002/84.3	0.982 (0.577-1.671)	0.000/88.7
MIX	REM	3 (3266/4914)	0.831 (0.711-0.971)	0.617/0.0	1.000 (0.910-1.099)	0.544/0.0	0.831 (0.716-0.964)	0.785/0.0	0.965 (0.883-1.055)	0.454/0.0	0.946 (0.885-1.010)	0.787/0.0
	FEM		0.831 (0.711-0.971)	0.617/0.0	1.000 (0.910-1.099)	0.544/0.0	0.831 (0.716-0.964)	0.785/0.0	0.965 (0.883-1.055)	0.454/0.0	0.946 (0.885-1.010)	0.787/0.0
**Source of control**												
HB	REM	37 (6828/10050)	1.129 (0.906-1.408)	0.000/49.0	1.086 (0.965-1.222)	0.000/59.8	1.087 (0.894-1.322)	0.007/40.4	1.100 (0.973-1.243)	0.000/65.8	1.019 (0.972-1.068)	0.422/2.8
	FEM		1.102 (0.965-1.257)	0.000/49.0			1.079 (0.950-1.225)	0.007/40.4			1.018 (0.972-1.065)	0.422/2.8
PB	REM	23 (11275/16920)	0.886 (0.793-0.991)	0.122/26.3	0.988 (0.939-1.040)	0.603/0.0	**0.890 (0.803-0.987)**	**0.161/22.7**	0.970 (0.922-1.020)	0.415/3.3	0.958 (0.916-1.002)	0.175/21.5
	FEM		**0.895 (0.822-0.976)**	**0.122/26.3**	0.988 (0.939-1.040)	0.603/0.0	**0.900 (0.829-0.977)**	**0.161/22.7**	0.970 (0.924-1.018)	0.415/3.3	**0.961 (0.926-0.998)**	**0.175/21.5**
**Type of control**												
Healthy	REM	23 (5707/7216)	0.973 (0.795-1.191)	0.030/39.0	1.125 (0.960-1.318)	0.000/72.0	0.937 (0.783-1.121)	0.086/30.2	1.114 (0.952-1.303)	0.000/74.3	1.059 (0.939-1.195)	0.000/73.1
	FEM		0.964 (0.843-1.101)	0.030/39.0			0.945 (0.832-1.074)	0.086/30.2				
Non-cancer	REM	37 (12396/19754)	0.958 (0.827-1.110)	0.001/47.8	0.995 (0.947-1.045)	0.683/0.0	0.956 (0.837-1.091)	0.006/41.1	0.987 (0.932-1.044)	0.143/20.1	0.984 (0.932-1.039)	0.005/41.8
	FEM		0.947 (0.869-1.032)	0.001/47.8	0.995 (0.948-1.045)	0.683/0.0	0.950 (0.875-1.031)	0.006/41.1	0.988 (0.943-1.035)	0.143/20.1	0.983 (0.948-1.019)	0.005/41.8
**HWE and Quality score > 12**												
**Overall**	REM	39 (15175/21511)	0.908 (0.809-1.020)	0.013/36.5	0.992 (0.948-1.037)	0.921/0.0	0.912 (0.821-1.012)	0.046/29.4	0.978 (0.937-1.021)	0.500/0.0	0.968 (0.927-1.010)	0.061/27.3
	FEM		**0.909 (0.840-0.985)**	**0.013/36.5**	0.991 (0.948-1.037)	0.921/0.0	**0.912 (0.845-0.985)**	**0.046/29.4**	0.978 (0.937-1.020)	0.500/0.0	0.969 (0.937-1.002)	0.061/27.3
**Ethnicity**												
Asian	REM	14 (3765/4875)	0.997 (0.725-1.372)	0.106/33.6	0.986 (0.898-1.083)	0.910/0.0	1.010 (0.743-1.371)	0.134/30.3	0.991 (0.905-1.085)	0.679/0.0	0.993 (0.913-1.080)	0.319/12.2
	FEM				0.985 (0.897-1.082)	0.910/0.0	1.039 (0.823-1.311)	0.134/30.3	0.990 (0.904-1.084)	0.679/0.0	0.997 (0.923-1.077)	0.319/12.2
Caucasian	REM	19 (7756/10698)	0.938 (0.812-1.082)	0.063/35.5	0.997 (0.936-1.062)	0.716/0.0	0.938 (0.823-1.068)	0.108/29.8	0.988 (0.930-1.049)	0.449/0.6	0.981 (0.926-1.038)	0.141/26.4
	FEM		0.951 (0.857-1.055)	0.063/35.5	0.997 (0.936-1.062)	0.716/0.0	0.951 (0.862-1.050)	0.108/29.8	0.988 (0.930-1.049)	0.449/0.6	0.982 (0.939-1.028)	0.141/26.4
MIX	REM	3 (3266/4914)	**0.831 (0.711-0.971)**	**0.617/0.0**	1.000 (0.910-1.099)	0.544/0.0	**0.831 (0.716-0.964)**	**0.785/0.0**	0.965 (0.883-1.055)	0.454/0.0	0.942 (0.880-1.008)	0.451/0.0
	FEM		**0.831 (0.711-0.971)**	**0.617/0.0**	1.000 (0.910-1.099)	0.544/0.0	**0.831 (0.716-0.964)**	**0.785/0.0**	0.965 (0.883-1.055)	0.454/0.0	0.942 (0.880-1.008)	0.451/0.0
**Source of control**												
HB	REM	20 (5080/7217)	0.979 (0.783-1.223)	0.046/37.7	0.982 (0.907-1.063)	0.955/0.0	0.997 (0.814-1.221)	0.094/30.8	0.985 (0.913-1.063)	0.668/0.0	0.987 (0.918-1.061)	0.187/21.6
	FEM		0.985 (0.845-1.147)	0.046/37.7	0.982 (0.907-1.063)	0.955/0.0	1.001 (0.864-1.159)	0.094/30.8	0.984 (0.912-1.062)	0.668/0.0	0.990 (0.932-1.052)	0.187/21.6
PB	REM	19 (10095/14294)	0.873 (0.776-0.995)	0.062/35.7	0.996 (0.943-1.052)	0.559/0.0	0.875 (0.780-0.981)	0.143/26.2	0.973 (0.917-1.033)	0.258/16.0	0.957 (0.907-1.009)	0.073/34.2
	FEM		**0.883 (0.805-0.969)**	**0.062/35.7**	0.996 (0.943-1.052)	0.559/0.0	**0.882 (0.807-0.964)**	**0.143/26.2**	0.974 (0.925-1.026)	0.258/16.0	**0.960 (0.923-0.999)**	**0.073/34.2**
**Type of control**												
Healthy	REM	12 (4382/4929)	0.882 (0.711-1.094)	0.121/33.7	1.004 (0.920-1.095)	0.548/0.0	0.874 (0.712-1.072)	0.131/32.5	0.983 (0.904-1.068)	0.452/0.0	0.974 (0.905-1.048)	0.291/15.6
	FEM		0.883 (0.757-1.029)	0.121/33.7	1.004 (0.920-1.095)	0.548/0.0	0.886 (0.765-1.027)	0.131/32.5	0.983 (0.905-1.068)	0.452/0.0	0.967 (0.907-1.031)	0.291/15.6
Non- cancer	REM	27 (10793/16582)	0.919 (0.797-1.059)	0.019/39.7	0.987 (0.937-1.041)	0.922/0.0	0.926 (0.818-1.050)	0.069/30.4	0.975 (0.927-1.026)	0.440/1.6	0.963 (0.913-1.016)	0.046/33.8
	FEM		0.919 (0.838-1.009)	0.019/39.7	0.987 (0.936-1.040)	0.922/0.0	0.922 (0.844-1.008)	0.069/30.4	0.976 (0.928-1.026)	0.440/1.6	0.970 (0.933-1.008)	0.046/33.8
**Egger’s test**												
***P***_***E***_			0.282		0.861		0.548		0.011		0.031	

HB, hospital-based studies; PB, population-based studies; REM, random effects model; FEM, fixed effects model. *P<0.05 was considered statistically significant.

Table 7 shows the results of subgroup analyses of *MTHFR A1298C* polymorphisms and CRC risk. It is worth noting that three genetic models (CC vs AA: *OR* =0.729, 95%*CI*=0.582-0.913(FEM); CC vs AA+AC: *OR* =0.751, 95%*CI*=0.604-0.933(FEM); and C vs A: *OR* =0.905, 95%*CI* =0.833-0.984 (FEM)) suggest that *MTHFR A1298C* reduces susceptibility in patients with rectal cancer, but in the colon cancer study only the recessive model showed similar results (CC vs AA+AC: *OR* =0.850, 95%*CI* =0.729-0.992 (FEM)). Moreover, the study did not find any statistical associations in stratified analyses based on gender, degree of differentiation, and tumour site. However, it should be noted that in subgroup analyses based on Dukes staging, the *MTHFR A1298C* polymorphism emerged as a susceptibility factor for stage A+B CRC (AC vs AA:*OR*=1.445,95%*CI*=1.059-1.972 (FEM)), while no statistical association could be found for stage C+D CRC. In the stratified analyses based on alcohol consumption, it was observed that both drinking and non-drinking subgroups demonstrated that *MTHFR A1298C* had a protective effect against CRC (non-drinking: CC vs. AA: *OR* =0.579, 95%*CI*=0.347-0.967 (FEM); CC vs. AA+AC: *OR* =0.610, 95%*CI* =0.376-0.989 (FEM). AC+CC vs. AA: *OR* =0.725, 95%*CI* =0.546-0.963 (FEM); C vs A: *OR* =0.311, 95%*CI* =0.189-0.512 (FEM); Drinking: CC vs AA: *OR* =0.249, 95%*CI*=0.087-0.711 (FEM); CC vs AA+ AC: *OR* =0.388, 95%*CI* =0.152-0.993 (FEM); C vs A: *OR* =0.744, 95%*CI* =0.623-0.889 (FEM)).

**Table 7:** Stratified analysis of association studies between polymorphisms at the *MTHFR A1298C* locus and risk of developing CRC.

Variable		CC vs.AA		AC vs. AA		CC vs. AA+AC		AC+CC vs. AA		C vs. A	
		OR (95% CI)	*Ph/I^2^(%)	OR (95% CI)	*Ph/I^2^ (%)	OR (95% CI)	*Ph/I^2^(%)	OR (95% CI)	*Ph/I^2^(%)	OR (95% CI)	*Ph/I^2^(%)
**Subgroupanalysis**											
**Location**											
Rectum 14 (2394/4911)	REM	**0.757(0.593-0.966)**	**0.411/3.6**	0.954(0.842-1.081)	0.277/16.1	**0.771(0.620-0.960)**	**0.591/0.0**	0.932(0.818-1.061)	0.177/25.7	0.923(0.828-1.029)	0.153/28.2
	FEM	**0.729(0.582-0.913)**	**0.411/3.6**	0.945(0.848-1.052)	0.277/16.1	**0.751(0.604-0.933)**	**0.591/0.0**	0.918(0.827-1.017)	0.177/25.7	**0.905(0.833-0.984)**	**0.153/28.2**
Colon 16 (4096/7258)	REM	0.925(0.665-1.286)	0.005/54.0	1.056(0.971-1.148)	0.451/0.1	0.907(0.676-1.219)	0.023/46.0	1.043(0.928-1.171)	0.079/35.4	1.008(0.904-1.124)	0.010/51.2
	FEM			1.055(0.970-1.147)	0.451/0.1	**0.850(0.729-0.992)**	**0.023/46.0**	1.025(0.947-1.111)	0.079/35.4		
**Gendering**											
Male 9 (2003/2828)	REM	1.007(0.629-1.611)	0.001/68.8	0.958(0.847-1.083)	0.534/0.0	1.027(0.661-1.596)	0.002/67.0	0.985(0.847-1.147)	0.145/34.1	1.009(0.864-1.178)	0.009/60.9
	FEM			0.958(0.847-1.083)	0.534/0.0			0.964(0.857-1.083)	0.145/34.1		
Female 9 (1564/2355)	REM	1.042(0.834-1.302)	0.496/0.0	0.961(0.833-1.108)	0.779/0.0	1.087(0.882-1.341)	0.446/0.0	0.982(0.859-1.122)	0.784/0.0	1.009(0.911-1.116)	0.620/0.0
	FEM	1.040(0.833-1.297)	0.496/0.0	0.961(0.834-1.107)	0.779/0.0	1.083(0.879-1.334)	0.446/0.0	0.982(0.860-1.122)	0.784/0.0	1.008(0.911-1.116)	0.620/0.0
Well + moderate 5 (633/1155)	REM	1.118(0.762- 1.641)	0.798/0.0	1.153(0.931- 1.427)	0.613/0.0	1.040(0.719- 1.504)	0.856/0.0	1.140(0.930-1.397)	0.565/0.0	1.091(0.930- 1.279)	0.588/0.0
	FEM	1.104(0.755- 1.614)	0.798/0.0	1.150(0.930- 1.423)	0.613/0.0	1.029(0.713- 1.484)	0.856/0.0	1.138(0.930-1.394)	0.565/0.0	1.089(0.929- 1.276)	0.588/0.0
Poor 5 (71/1155)	REM	0.752(0.244-2.318)	0.835/0.0	0.985(0.496-1.954)	0.217/30.7	0.797(0.282-2.251)	0.965/0.0	0.906(0.489-1.679)	0.265/23.4	0.906(0.595- 1.378)	0.452/0.0
	FEM	0.691(0.226-2.114)	0.835/0.0	0.994(0.595-1.660)	0.217/30.7	0.756(0.268- 2.128)	0.965/0.0	0.924(0.563-1.517)	0.265/23.4	0.877(0.580-1.327)	0.452/0.0
**Location**											
Proximal 4 (344/1543)	REM	1.291(0.770-2.163)	0.055/60.6	1.172(0.806-1.706)	0.172/40.0	1.230(0.555- 2.728)	0.083/55.0	1.180(0.796-1.751)	0.119/48.7	1.127(0.927-1.371)	0.081/55.4
	FEM			1.162(0.899-1.502)	0.172/40.0			1.171(0.915-1.499)	0.119/48.7		
Distal 4 (515/1543)	REM	1.357(0.822-2.239)	0.251/26.8	1.088(0.877-1.349)	0.764/0.0	1.325(0.811-2.165)	0.243/28.1	1.130(0.920-1.387)	0.677/0.0	1.132(0.961-1.335)	0.422/0.0
	FEM	1.342(0.887-2.031)	0.251/26.8	1.087(0.876-1.348)	0.764/0.0	1.305(0.874-1.948)	0.243/28.1	1.128(0.919-1.386)	0.677/0.0	1.129(0.958-1.331)	0.422/0.0
**Dukes**											
A+B 3 (232/817)	REM	1.392(0.347-5.591)	0.043/68.2	**1.467(1.005-2.142)**	**0.257/26.4**	1.101(0.330- 3.667)	0.083/59.8	1.452(0.900-2.341)	0.110/54.8	1.277(0.824- 1.977)	0.050/66.7
	FEM			**1.445(1.059-1.972)**	**0.257/26.4**						
C+D 3 (233/817)	REM	1.124(0.612-2.063)	0.594/0.0	0.953(0.691-1.314)	0.975/0.0	1.148(0.639- 2.064)	0.604/0.0	0.971(0.714-1.319)	0.874/0.0	1.002(0.785-1.279)	0.710/0.0
	FEM	1.091(0.601-1.980)	0.594/0.0	0.953(0.691-1.313)	0.975/0.0	1.117(0.628- 1.987)	0.604/0.0	0.970(0.714-1.317)	0.874/0.0	1.000(0.784-1.275)	0.710/0.0
**Drink**											
NON-drinker 3 (386/546)	REM	**0.580(0.347- 0.969)**	**0.415/0.0**	0.762(0.536-1.084)	0.288/19.6	**0.612(0.377-0.993)**	**0.412/0.0**	**0.730(0.549-0.971)**	**0.378/0.0**	0.415(0.160-1.076)	0.173/43.0
	FEM	**0.579(0.347- 0.967)**	**0.415/0.0**	0.766(0.569-1.032)	0.288/19.6	**0.610(0.376-0.989)**	**0.412/0.0**	**0.725(0.546-0.963)**	**0.378/0.0**	**0.311(0.189-0.512)**	**0.173/43.0**
Drinker 3 (160/282)	REM	**0.271(0.095-0.770)**	**0.803/0.0**	0.767(0.319-1.847)	0.017/75.4	0.454(0.171-1.205)	0.528/0.0	0.696(0.313-1.549)	0.028/72.1	**0.749(0.617-0.909)**	**0.371/7.7**
	FEM	**0.249(0.087-0.711)**	**0.803/0.0**			**0.388(0.152-0.993)**	**0.528/0.0**			**0.744(0.623-0.889)**	**0.371/7.7**

HB, hospital-based studies; PB, population-based studies; REM, random effects model; FEM, fixed effects model. *P<0.05 was considered statistically significant.

### Heterogeneity and sensitivity analyses

Meta-regression analyses were conducted to investigate potential sources of heterogeneity in the overall and subgroup analyses. The sources of heterogeneity for *MTHFR C677T* were race (TT vs.CC:*P* = 0.004;CT+TT vs.CC: *P* = 0.012; TT vs. CC+CT: *P* = 0.010; T vs. C: *P* = 0.002), type of control (TT vs. CC+CT: *P* = 0.032), and *HWE* (T vs. C: *P* = 0.043),while quality score (AC vs. AA: *P* = 0.045) was identified as a source of heterogeneity for the *MTHFR A1298C* polymorphism and CRC risk.

**Fig 4:**
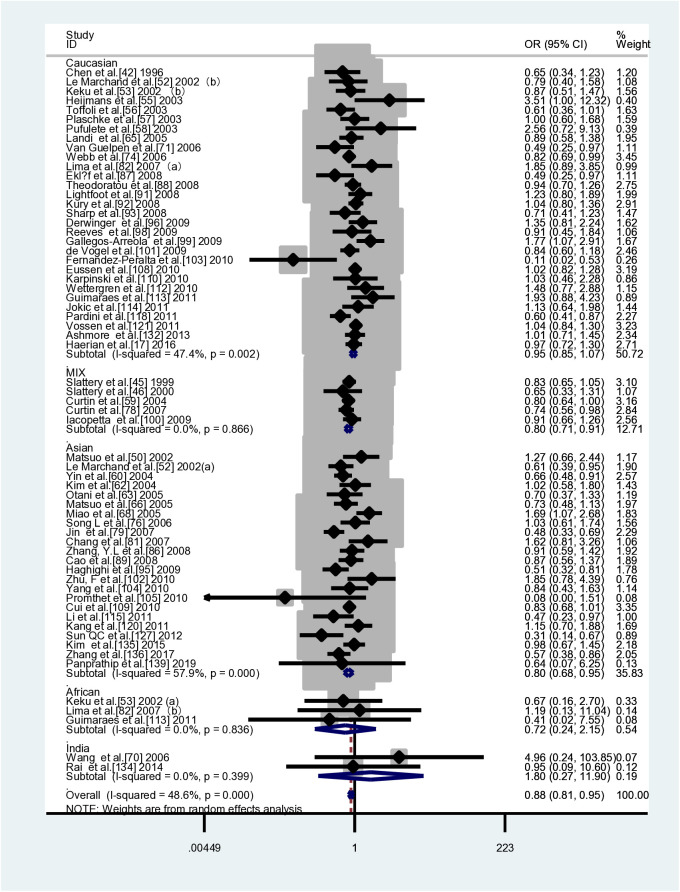
Forest plot of sensitivity analysis of *MTHFR C677T* polymorphism associated with CRC susceptibility sensitivity analyses for TT vs. CC. (Quality score >12 and consistent with HWE).

**Fig 5:**
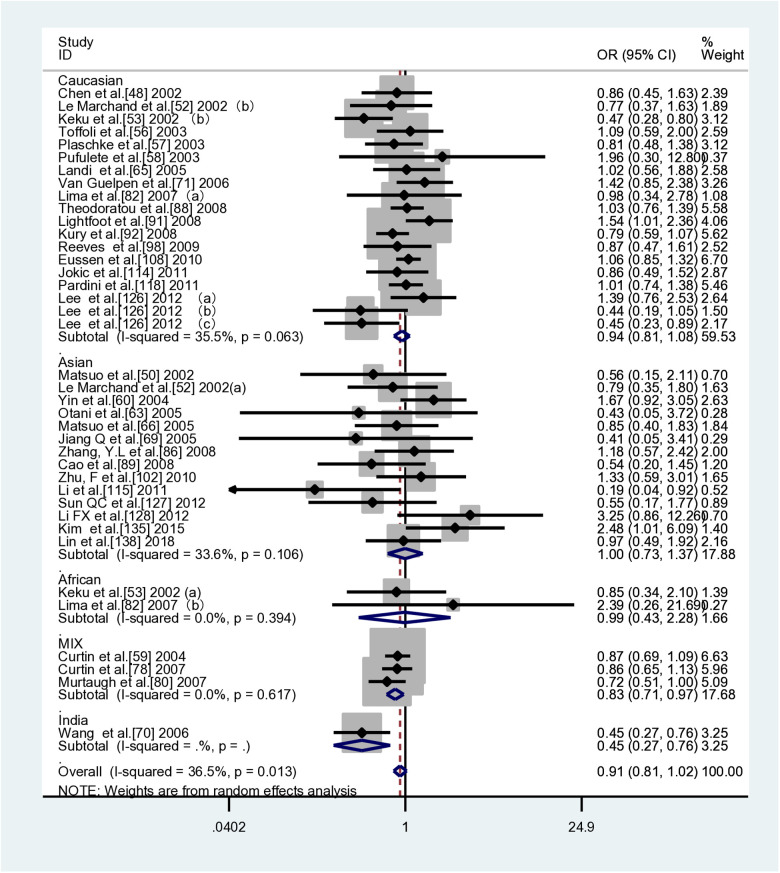
Sensitivity analysis of MTHFR A1298C polymorphism and CRC risk (CC vs. AA, HWE compliant).

Sensitivity analyses were performed to assess the stability of the included studies. When only high-quality and HWE-matched studies were included, there were differences in the results between the *MTHFR C677T* and A1298C polymorphisms and the risk of CRC, with the corresponding combined ORs being significantly affected as follows: Overall, four genetic models suggested that the *MTHFR C677T* polymorphism was a protective factor for CRC (TT vs. CC: *OR*=0.866, 95% *CI*=0.822-0.913 (FEM); TT vs. CC+CT: *OR*=0.879,95% *CI*=0.837-0.922 (FEM); CT+TT vs. CC: *OR*=0.966,95% *CI*=0.935-0.997 (FEM); T vs. C: *OR*=0.953, 95% *CI*= 0.917-0.990).In particular, when analysed by ethnic stratification, the results showed that the *MTHFR C677T* gene polymorphism reduced the risk of CRC in Asian, mixed-race individuals, which is consistent with previous results, but unfortunately,sensitivity analyses were not performed for Indian race due to the small number of studies on Indian race.In addition, the findings remained consistent when subgroup analyses were performed according to the source and type of control. Taken together, this suggests that the findings on *MTHFR C677T* and CRC susceptibility are stable. For *MTHFR A1298C*, when restricted to high-quality and HWE-compliant studies, the overall data suggest that the *MTHFR A1298C* polymorphism is a protective factor for CRC (CC vs. AA: OR = 0.909, 95% CI = 0.840-0.985 (FEM); CC vs. AA+AC: OR = 0.912, 95% CI = 0.845-0. 985 (FEM)), and race-based subgroup analyses showed additive and recessive models for mixed races (CC vs. AA: OR = 0.831, 95% CI= 0.711-0.971 (FEM); CC vs. AA+AC: OR = 0.831, 95% CI= 0.716-0.964 (FEM)), indicating that the *MTHFR A1298C* was able to reduce susceptibility to CRC. This is a significant difference from previous results, which were not stable enough. More details of the results of the sensitivity analyses are shown in Tables 4 and 6, and the forest plot results are shown in Figs 4 and 5.

### Publication bias

**Fig 6:**
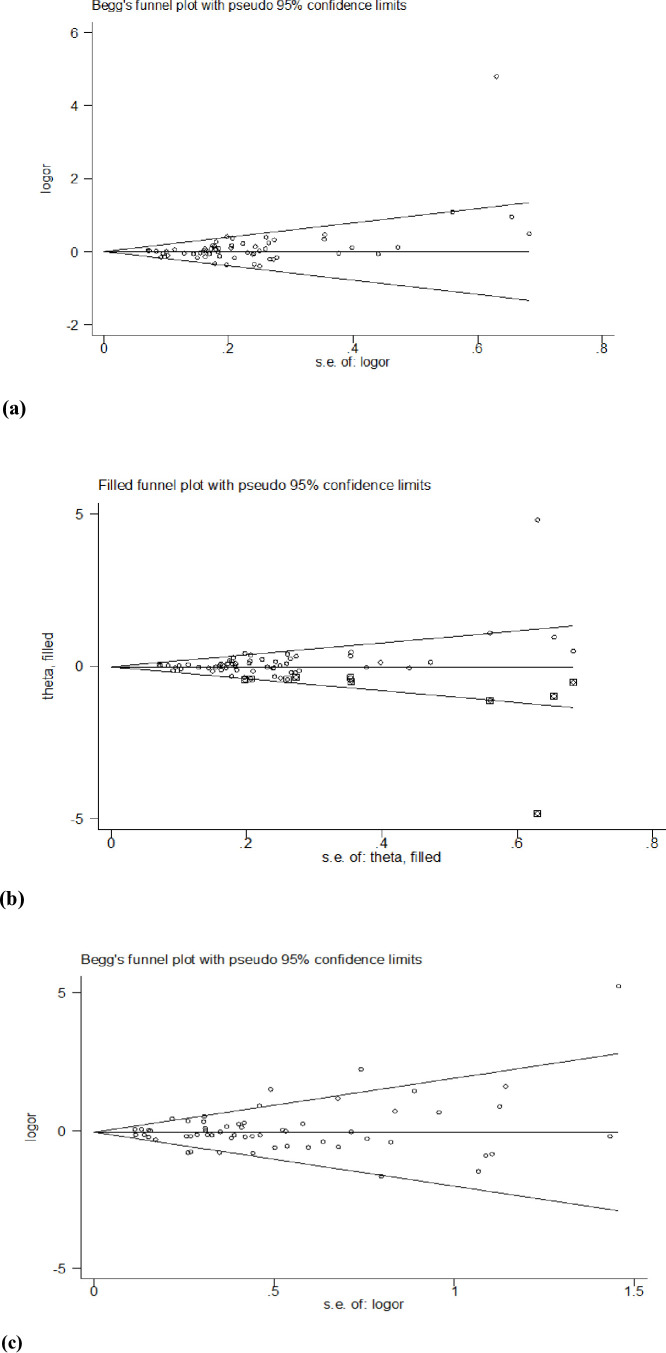
Begg’s funnel plot of *MTHFR* gene polymorphisms on CRC susceptibility in the general population. (a): *MTHFR C677T* (CT VS CC); Egger’s assay: PE=0.008 < 0.01. (b): *MTHFR C677T* (CT VS CC); Using the non-parametric “trim and fill” method to adjust the publication offset. (c): *MTHFR A1298C* (CC vs AA); Egger’s test: PE > 0.01.

In the present study, Begg funnel plot and Egger’s test were used to evaluate publication bias, and the results were shown as follows: for *MTHFR C677T*, the Begg funnel plot (Fig 6a) was asymmetric, and the Egger’s test: CT vs CC: *PE*=0.008<0.01 (the Egger’s test results for the other models are shown in Table 4), which suggested that the existence of publication bias was considered, and then the results were adjusted using the non-parametric “trimming The results were then adjusted using a non-parametric “trim and fill” approach (Fig 6b), suggesting that the current publication bias did not affect the results. For *MTHFR A1298C*, the funnel plot (Fig 6c) was approximately symmetrical for all models, with Egger’s test results >0.01 (Table 6), suggesting no publication bias.

### Credibility of the identified genetic associations

The study’s credibility was assessed using FPRP and BFDP. The criteria were as follows: Highly credible association: (1) statistically significant association of at least two genetic models (p-value of Z-test < 0.05); (2) *I*^*2*^ < 50%; (3) statistical efficacy > 80%; and (4) FPRP < 0.2 and BFDP < 0.8. Less credible deterministic associations: (1) statistically significant association of at least one genetic model, *p* < 0.05; (2) Statistical power between 50-79%, or FPRP > 0.2 or *I*^*2*^ > 50%. Otherwise, they were classified as “null” or “negative” associations. Table 8 displays the confidence assessment results of the association between the MTHFR *C677T* and *A1298C* polymorphisms with CRC susceptibility. In this meta-analysis, the statistical associations between the *MTHFR C677T* polymorphism and CRC susceptibility studies were classified as ‘false-positive results with low confidence’ after the credibility assessment. Meanwhile, the associations between the *MTHFR A1298C* polymorphism and CRC susceptibility studies were classified as ‘unreliable results.’

**Table 8:** Credibility assessment of this meta-analysis.

Variable	Genetic model	*OR* (*95% CI*)	*I*^*2*^ (%)	Statistical effect	Credibility assessment	
					Prior probability <0.001	
					FPRP	BFDP
***MTHFR C677T***						
Overall	TT vs.CC	0.888(0.822-0.959)	57.1	1.000	0.712	0.993
	TT vs. CC+CT	0.891(0.831-0.956)	54.2	1.000	0.568	0.990
Asian	TT vs.CC	0.783(0.669-0.917)	65.7	0.977	0.711	0.987
	TT vs. CC+CT	0.811(0.708-0.929)	61.7	0.998	0.715	0.989
	T vs. C	0.906(0.836-0.981)	72.5	1.000	0.937	0.999
India	CT+TT vs. CC	1.329(1.033-1.709)	0.0	0.827	0.970	0.998
	T vs. C	1.336(1.065-1.675)	0.0	0.842	0.935	0.996
MIX	TT vs.CC	0.831(0.746-0.926)	0.0	1.000	0.445	0.975
	TT vs. CC+CT	0.824(0.743-0.914)	0.0	1.000	0.201	0.933
	T vs. C	0.946(0.902-0.993)	0.0	1.000	0.961	0.999
PB	TT vs.CC	0.850(0.804-0.898)	25.3	1.000	0.000	0.001
	TT vs. CC+CT	0.845(0.802-0.890)	33.0	1.000	0.000	0.000
	T vs. C	0.954(0.931-0.978)	16.9	1.000	0.169	0.978
Non-cancer	TT vs.CC	0.834(0.792-0.877)	47.4	1.000	0.000	0.000
	TT vs. CC+CT	0.834(0.795-0.875)	36.4	1.000	0.000	0.000
	T vs. C	0.929(0.892-0.968)	58.4	1.000	0.309	0.983
**HWE and Quality score > 12**						
Overall	TT vs.CC	0.866(0.822-0.913)	48.6	1.000	0.000	0.014
	TT vs. CC+CT	0.879(0.837-0.922)	44.0	1.000	0.000	0.019
	CT+TT vs. CC	0.966(0.935-0.997)	39.8	1.000	0.970	1.000
	T vs. C	0.953(0.917-0.990)	52.1	1.000	0.930	0.999
Asian	TT vs.CC	0.804(0.683-0.945)	57.9	0.988	0.892	0.996
	CTvs.CC	0.922(0.861-0.986)	42.0	1.000	0.946	0.999
	TT vs. CC+CT	0.836(0.768-0.911)	47.4	1.000	0.042	0.761
	CT+TT vs. CC	0.893(0.805-0.990)	55.0	1.000	0.969	0.999
	T vs. C	0.905(0.836-0.981)	63.3	1.000	0.938	0.999
MIX	TT vs.CC	0.804(0.708-0.913)	0.0	0.998	0.435	0.970
	TT vs. CC+CT	0.802(0.710-0.905)	0.0	0.999	0.256	0.942
	T vs. C	0.935(0.885-0.988)	0.0	1.000	0.944	0.999
	TT vs. CC+CT	0.912(0.843-0.986)	47.9	1.000	0.954	0.999
HB	TT vs.CC	0.856(0.801-0.914)	26.8	1.000	0.003	0.262
PB	TT vs. CC+CT	0.858(0.807-0.914)	37.0	1.000	0.002	0.187
	T vs. C	0.953(0.925-0.982)	20.2	1.000	0.622	0.996
Non-cancer	TT vs.CC	0.837(0.788-0.888)	30.8	1.000	0.000	0.001
	TT vs. CC+CT	0.844(0.798-0.893)	18.1	1.000	0.000	0.001
	CT+TT vs. CC	0.961(0.927-0.996)	38.2	1.000	0.967	1.000
**Subgroup Analysis**						
Tumor Location						
Colon	TT vs. CC+CT	0.833(0.698-0.994)	68.1	0.993	0.977	0.999
Differentiation						
Poor	TT vs.CC	0.592(0.380-0.923)	0.0	0.300	0.986	0.996
Non-mucinous	TT vs.CC	0.503(0.281-0.899)	51.2	0.171	0.992	0.996
	CTvs.CC	0.775(0.627-0.958)	0.0	0.918	0.953	0.997
	TT vs. CC+CT	0.509(0.377-0.687)	31.7	0.039	0.207	0.305
	CT+TT vs. CC	0.685(0.561-0.838)	36.9	0.604	0.280	0.888
	T vs. C	0.729(0.552-0.964)	53.6	0.735	0.973	0.998
Location						
Proximal	CTvs.CC	1.251(1.003-1.560)	71.3	0.946	0.980	0.999
Distal	TT vs.CC	0.853(0.729-0.997)	0.0	0.999	0.979	0.999
Dukes						
A	TT vs.CC	0.434(0.234-0.808)	18.4	0.088	0.990	0.993
	TT vs. CC+CT	0.505(0.282-0.907)	0.0	0.176	0.992	0.996
C	TT vs.CC	0.601(0.428-0.844)	11.7	0.275	0.923	0.985
	TT vs. CC+CT	0.646(0.468-0.892)	8.9	0.424	0.949	0.993
	T vs. C	0.821(0.712-0.945)	45.6	0.998	0.857	0.995
A+B	TT vs. CC+CT	0.649(0.496-0.850)	45.7	0.423	0.799	0.976
Drink						
NON-drinker	TT vs.CC	0.459(0.339-0.622)	30.0	0.008	0.060	0.030
	TT vs. CC+CT	0.524(0.395-0.697)	0.0	0.049	0.155	0.280
	CT+TT vs. CC	0.675(0.485-0.939)	59.8	0.529	0.974	0.997
	T vs. C	0.823(0.731- 0.926)	0.0	1.000	0.546	0.981
Drinker	TT vs.CC	0.432(0.258-0.725)	16.6	0.050	0.967	0.974
	TT vs. CC+CT	0.511(0.319-0.821)	27.4	0.136	0.976	0.990
	CT+TT vs. CC	0.655(0.476-0.901)	0.0	0.457	0.953	0.994
	T vs. C	0.812(0.666-0.991)	0.0	0.974	0.976	0.999
***MTHFRA1298C***						
PB	CC vs.AA	0.895(0.822-0.976)	26.3	1.000	0.924	0.998
	CA vs. AA	0.988(0.939-1.040)	0.0	1.000	0.998	1.000
	CC vs. AA+AC	0.900(0.829-0.977)	22.7	1.000	0.922	0.998
	C vs. A	0.961(0.926-0.998)	21.5	1.000	0.975	1.000
**HWE and Quality score > 12**						
Overall	CC vs.AA	0.909(0.840-0.985)	36.5	1.000	0.952	0.999
	CC vs. AA+AC	0.912(0.845-0.985)	29.4	1.000	0.950	0.999
PB	CC vs.AA	0.883(0.805-0.969)	35.7	1.000	0.897	0.997
	CC vs. AA+AC	0.882(0.807-0.964)	26.2	1.000	0.849	0.996
	C vs. A	0.960(0.923-0.999)	34.2	1.000	0.978	1.000
**Subgroup Analysis**						
Tumor Location						
Rectum	CC vs.AA	0.729(0.582- 0.913)	3.6	0.782	0.883	0.992
	CC vs. AA+AC	0.751(0.604-0.933)	0.0	0.859	0.919	0.995
	C vs. A	0.905(0.833-0.984)	28.2	1.000	0.951	0.999
Colon	CC vs. AA+AC	0.850(0.729-0.992)	46.0	0.999	0.975	0.999
Dukes						
A+B	CA vs. AA	1.445(1.059-1.972)	26.4	0.593	0.972	0.997
Drink						
NON-drinker	CC vs.AA	0.579(0.347- 0.967)	0.0	0.295	0.992	0.997
	CC vs. AA+AC	0.610(0.376-0.989)	0.0	0.359	0.992	0.998
	AC +CC vs.AA	0.725(0.546-0.963)	0.0	0.719	0.973	0.997
	C vs. A	0.311(0.189-0.512)	43.0	0.001	0.763	0.278
Drinker	CC vs.AA	0.249(0.087-0.711)	0.0	0.033	0.997	0.996
	CC vs. AA+AC	0.388(0.152-0.993)	0.0	0.129	0.997	0.998
	C vs. A	0.744(0.623-0.889)	7.7	0.886	0.561	0.973

HB, hospital-based studies; PB, population-based studies.

## Discussion

The development of CRC is influenced by genetic, epigenetic, dietary, and environmental factors. Research has shown that folate status, methionine levels, and alcohol consumption are all risk factors for the association between *MTHFR* polymorphisms and CRC [78]. Genetics is a key determinant in the development of CRC [153], and polymorphisms in cancer-related genes may influence inter-individual susceptibility to CRC [154]. Various studies have identified single nucleotide polymorphisms (SNPs) in the *VEGF*, *CYP1B1*, *P53*, and *NOD2* genes as modifiers of CRC risk across different ethnicities. However, the association between polymorphisms in folate-related genes and susceptibility to CRC remains inconclusive. *MTHFR* is a key enzyme in folate metabolism that converts 5,10-methylenetetrahydrofolate to 5-methyltetrahydrofolate. This conversion may play a significant role in CRC carcinogenesis. 5,10-methylenetetrahydrofolate is closely related to thymidylate synthesis, while 5-methyltetrahydrofolate promotes methionine synthesis and SAM-mediated methylation [30]. The correct operation of this metabolic pathway is crucial for maintaining normal DNA methylation, nucleotide resynthesis, and DNA repair [155]. Polymorphisms in the *MTHFR* gene can cause changes in enzyme activity, resulting in low levels of folate and high levels of homocysteine. This microenvironment results in DNA hypomethylation in vivo, which impacts DNA synthesis, repair, and ultimately DNA stability, as well as the expression of proto-oncogenes and oncogenes. These factors are closely linked to tumour susceptibility [156]. The role of the *MTHFR* gene in CRC aetiology has been extensively studied in recent years. However, no consensus has been reached, possibly due to variations in study design, geographic region, ethnicity, and dietary habits. Furthermore, there are notable variations in the incidence and mortality rates of CRC, as well as recent epidemiological trends, across different countries and regions of the world. These differences can be attributed, in part, to variations in risk factor exposure, demographic characteristics, and genetic factors (including mutations), and their impact on treatment outcomes and responses in different geographic regions and populations [157–159]. Additionally, the epidemiological characteristics of proximal and distal colon cancers differ across age and gender and are subject to multiple confounding factors. Therefore, to provide a more precise assessment of the association between the *MTHFR C677T* and *A1298C* polymorphisms with CRC susceptibility, it is necessary to conduct more detailed stratified analyses of confounding factors such as race, tumour site, and gender.

There have been 26 and 13 previous meta-analyses assessing the link between *MTHFR C677T* and *A1298C* polymorphisms and susceptibility to CRC, respectively. The most recent publications were in 2017 [15] and 2015 [41], and the studies included were mainly conducted before 2016 and 2014. The latest study included in our analysis was from 2021. It should be noted that this meta-analysis has the largest sample size to date, with included studies having maximum sample sizes of 106 and 60, compared to previous meta-analyses with maximum sample sizes of 86 and 46. When reviewing published meta-analyses, it is important to note that there can be significant variability in study results. Houlston et al. conducted a meta-analysis in 2001 to explore the relationship between *MTHFR C677T* and CRC susceptibility. The study found that the *MTHFR C677T* polymorphism was a protective factor for CRC.

However, a meta-analysis conducted by Chen et al. in 2005 found no significant correlation between the two variables. This lack of correlation may be attributed to the small sample size of included studies. In 2006, two studies [37,38] investigated the relationship between two SNP loci of *MTHFR* (*C677T* and *A1298C*) and CRC susceptibility for the first time. Both studies concluded that *C677T* was associated with CRC susceptibility. However, Yu et al. suggested that *1298CC* might also have a protective effect against CRC, and Sun et al. found no statistically significant relationship between *A1298C* and CRC. In a study conducted by Hubner et al. in the same year, it was found that the *677TT* genotype was associated with a lower risk of CRC compared to the pure CC genotype. However, it is important to note that only English articles were included in this study. Since then, 14 meta-analyses [16,20–23,25,27–31,33–35] have shown a negative relationship between *MTHFR C677T* and CRC susceptibility, while 5 studies [15,17,19,24,32] have concluded that there is no correlation between the two. Two articles [18,26] have shown that *MTHFR C677T* is a risk factor for CRC. Four studies [30,35,37,41] have found a negative relationship between *MTHFR A1298C* and CRC, while five studies [22,25,31,32,38] have found no statistical relationship. It is worth noting that Haerian BS et al. concluded that *MTHFR A1298C* is a risk factor for CRC, possibly due to genetic modelling errors.

After reviewing the meta-analyses published on the link between polymorphisms in the *MTHFR* gene and susceptibility to CRC, we identified several shortcomings. Firstly, only a few studies conducted sensitivity analyses on the meta-analysis results. Sensitivity analyses help to identify which studies had a significant impact on the meta-analysis results, thus providing a better understanding of the robustness and limitations of the results. Second, few studies have explored sources of heterogeneity using meta-regression equations or subgroup analyses. Finding sources of heterogeneity can help us better understand the heterogeneity of meta-analysis results and identify which factors may have had a significant impact on the meta-analysis results, which can help us better interpret the meta-analysis results and present more accurate conclusions. In addition, only 10 studies were tested for *HWE*, which is needed for reliable genetic association studies in meta-analyses. If the control group does not meet the *HWE* criteria, there may be selection bias or genotyping errors that could lead to misleading results.There was variability in the establishment of genetic models among studies, with only 10 out of 14 meta-analyses using five genetic models, which could lead to false-negative results. Additionally, Teng [26] et al. and Haerian BS [18] et al. had incorrectly established genetic models in their studies, resulting in significant variability from the results of other meta-analyses. It should be noted that previous meta-analyses did not find statistically significant associations for the probability of false positive reports. Therefore, their results may have been false positives or false negatives.

It is important to acknowledge the limitations of this meta-analysis. Firstly, it only includes published articles, which may introduce publication bias. This means that the results may not be representative of all studies and may be skewed towards those with significant results. Therefore, some research results may have been omitted. (2) This meta-analysis only includes studies published in English or Chinese, which may introduce publication bias by excluding non-significant or negative results in other languages. To address this, we used Begg’s funnel plot and Egger’s test. (3) The meta-analysis included studies of varying quality and sample size. For example, only four studies on the *MTHFR C677T* polymorphism and CRC risk in African populations and six studies on CRC in Indian populations were accounted for. Similarly, only four studies on the *MTHFR A1298C* polymorphism and CRC susceptibility in African and Indian populations, respectively, were included, along with three others. This raises the possibility that there may not be enough statistical power to explore true associations. (4) Some studies selected controls from non-cancer patients undergoing colonoscopy, while others only selected controls from asymptomatic populations. This may lead to misclassification bias as potential cancer cases may not be excluded from the control group. (5) Due to insufficient data, this meta-analysis did not adequately elucidate gene-gene and gene-environment interactions.

Despite the limitations mentioned above, the meta-analysis has several advantages over previous studies. Firstly, the sample size was significantly increased. This study represents the largest meta-analysis to date exploring the relationship between *MTHFR* polymorphisms and CRC susceptibility, and the data collected are much more comprehensive, providing more reliable evidence for the association between *MTHFR* polymorphisms and CRC risk. (2) Previous meta-analyses have assessed the association with CRC susceptibility without systematically evaluating colon and rectal cancers. Our study demonstrated that *MTHFR C677T* reduced susceptibility to colon cancer, but not significantly for rectal cancer. However, the *MTHFR A1298C* polymorphism was inversely associated with the risk of both colon and rectal cancers. This finding may be related to different oncogenic mechanisms. (3) The meta-analysis was based on unadjusted ORs and 95% CIs. To account for the effects of multiple confounding factors, such as age, sex, dietary habits (including alcohol consumption, smoking, and folate intake), tumour location, degree of differentiation, TNM stage, and other environmental factors, we conducted a more comprehensive subgroup analysis. This allowed us to explore the relationship between *MTHFR* polymorphisms and susceptibility to colorectal cancer. (4) Quality scores and Hardy-Weinberg equilibrium (HWE) tests were rerun for all included studies to exclude the influence of low-quality and HWE-deviating studies on the results. This improves the reliability and accuracy of the meta-analysis. (5) The sources of heterogeneity were explored using meta-regression analysis and subgroup analysis. (6) False-positive report probability (FPRP) and Bayesian false discovery probability (BFDP) were applied to assess associations and avoid misleading false-positive results.

## Conclusion

Overall, the study indicates that the *MTHFR C677T* gene polymorphism decreases the risk of colorectal cancer in Asian and mixed-race populations, but increases the risk in the Indian ethnic group. Furthermore, *MTHFR A1298C* may play a protective role in the development of CRC, although additional investigation is required to determine the specific mechanism of action and influencing factors. The findings presented here not only enhance the biological understanding of gene polymorphisms in CRC, but also offer a new perspective on the potential application of targeted induced mutations in the field of tumour diagnosis and treatment. By combining the confounding factors, an integrated scoring system can be established, which can provide a more comprehensive and accurate reference for individualized diagnosis and treatment of CRC.

## Supporting Information

S1 Table Scale for quality assessment of molecular association studies of gastric cancer. (DOCX)

S2 Table plosone-checklist. (DOCX)

S3 Table PRISMA 2020 checklist. (DOCX)

## Abbreviations

*MTHFR*: Methylenetetrahydrofolate reductase
CRC: Colorectal cancer
*OR*: Odds ratio
*95%CI*: 95% confidence interval
*HWE*: Hardy–Weinberg equilibrium
*HWD*: Hardy-Weinberg Disequilibrium
REM: Random effects model
FEM: Fixed effects model
FPRP: false-positive report probabilities
BFDP: Bayesian false discovery probability
PRISMA: Preferred Reporting Items for Systematic Review and Meta-Analyses
*SNP*: Single nucleotide polymorphisms
